# Increased levels of lagging strand polymerase α in an adult stem cell lineage affect replication-coupled histone incorporation

**DOI:** 10.1126/sciadv.adu6799

**Published:** 2025-02-28

**Authors:** Brendon E. M. Davis, Jonathan Snedeker, Rajesh Ranjan, Matthew Wooten, Savannah Sáde Barton, Joshua Blundon, Xin Chen

**Affiliations:** ^1^ Department of Biology, The Johns Hopkins University, Baltimore, MD 21218, USA.; ^2^ Howard Hughes Medical Institute, Department of Biology, The Johns Hopkins University, 3400 North Charles Street, Baltimore, MD 21218, USA.

## Abstract

Stem cells display asymmetric histone inheritance, while nonstem progenitor cells exhibit symmetric patterns in the *Drosophila* male germ line. Here, we report that components involved in lagging strand synthesis, DNA polymerases α and δ, have substantially reduced levels in stem cells compared to progenitor cells, and this promotes local asymmetry of parental histone incorporation at the replication fork. Compromising Polα genetically induces the local replication-coupled histone incorporation pattern in progenitor cells to resemble that in stem cells, seen by both nuclear localization patterns and chromatin fibers. This is recapitulated using a Polα inhibitor in a concentration-dependent manner. The local old versus new histone asymmetry is comparable between stem cells and progenitor cells at both S phase and M phase. Together, these results indicate that developmentally programmed expression of key DNA replication components is important to shape stem cell chromatin. Furthermore, manipulating one crucial DNA replication component can induce replication-coupled histone dynamics in nonstem cells to resemble those in stem cells.

## Teaser

Replication-coupled histone incorporation is regulated by DNA polymerase levels.

## INTRODUCTION

Regarding metazoan development, an outstanding question is how cells take on distinct fates and have diverse functions although they derive from one zygote. Cell fate is determined by selectively expressing a subset of the genome at the proper time, in the right place, and at the precise level. The unique gene expression program for each cell type is typically regulated by epigenetic mechanisms, which refer to chromatin changes without alteration of the DNA sequences (*1*–*3*). Epigenetic mechanisms comprise DNA methylation, histone modifications, histone variants, as well as noncoding RNAs, among others. However, except for DNA methylation, how the epigenetic information is transferred through the active cell cycle in multicellular organisms remains largely unclear but has recently become an area of major research interest (*4*, *5*). Notably, these mechanisms could not only be responsible for maintaining epigenetic memory but also allow for epigenetic changes to diversify cell fates, which are essential for development, homeostasis, and regeneration (*6*–*8*). One paradigmatic model to study cell fate decision is asymmetric cell division (ACD), through which one mother cell gives rise to two distinct daughter cells. Upon ACD, the genetic codes inherited by the two daughter cells are identical except in special cases (*9*), whereas their epigenetic information can vary, allowing them to appear and function differently [reviewed in (*10*–*14*)].

To investigate the histone inheritance pattern in ACD, a tag-switch strategy to differentially label preexisting (old) versus newly synthesized (new) histones has been developed and used to study the *Drosophila* adult stem cell systems. These studies reveal that old histones are selectively retained in the self-renewing stem cell, whereas new histones are enriched in the differentiating daughter cell during ACDs of male germline stem cells (GSCs) (*15*, *16*) and intestinal stem cells (*17*). Moreover, the asymmetric histone pattern is specific to local regions in female GSCs (*18*) and Wnt3a-induced asymmetrically dividing mouse embryonic stem cells (*19*). Notably, in the male germline lineage, old and new histones are inherited symmetrically during the symmetric divisions of the progenitor spermatogonial cells (SGs). Asymmetric histone inheritance has been proposed to involve a process with at least three steps: First, old and new histones are asymmetrically incorporated on the replicative sister chromatids, attributed by both strand-specific incorporation and biased replication fork movement, including increased unidirectional and asymmetric bidirectional fork progression in early-stage germ cells (*16*, *20*). Then, the epigenetically distinct sister chromatids are differentially recognized and segregated during mitosis (*21*), leading to distinct “read-outs” in the resulting two daughter cells, such as their asynchronous S phase initiation (*22*) and distinct interchromosomal interactions at a key “stemness” gene (*23*).

Despite this knowledge, two crucial questions still remain: First, what are the precise molecular mechanisms that ensure asymmetric histone incorporation at the individual replication forks? A series of studies has extensively explored the roles of DNA replication components in establishing the epigenomes in unicellular organisms, such as yeast (*24*–*28*), and symmetrically dividing cells, such as cultured mouse embryonic stem cells (*29*–*35*) and human cell lines (*36*, *37*). These studies focus on how epigenetic information can be equally partitioned between sister chromatids and inherited symmetrically by the daughter cells [reviewed in (*4*, *38*–*41*)]. Little is known about this process in asymmetrically dividing cells in multicellular organisms. Studies in mouse development demonstrate that asymmetric inheritance of the histone modification H3R26me2 (*42*) or maternal chromosome-bound H3.3 and H3K9me2 (*43*) are essential for early embryogenesis, in contrast to the negative effects of asymmetric histone inheritance in yeast (*44*–*46*) and mouse embryonic stem cells (*31*, *32*, *34*, *35*), emphasizing the importance to study this phenomenon in an organism- and context-dependent manner. Second, how are these mechanisms regulated in a stage-specific manner within the same adult stem cell lineage, such that histone inheritance is asymmetric in stem cells (e.g., GSCs) but symmetric in progenitor cells (e.g., SGs)? Here, we used the *Drosophila* male germ line as a model system to address these questions.

## RESULTS

### Differential expression of the lagging strand-enriched replication components in GSCs

**Fig. 1.**
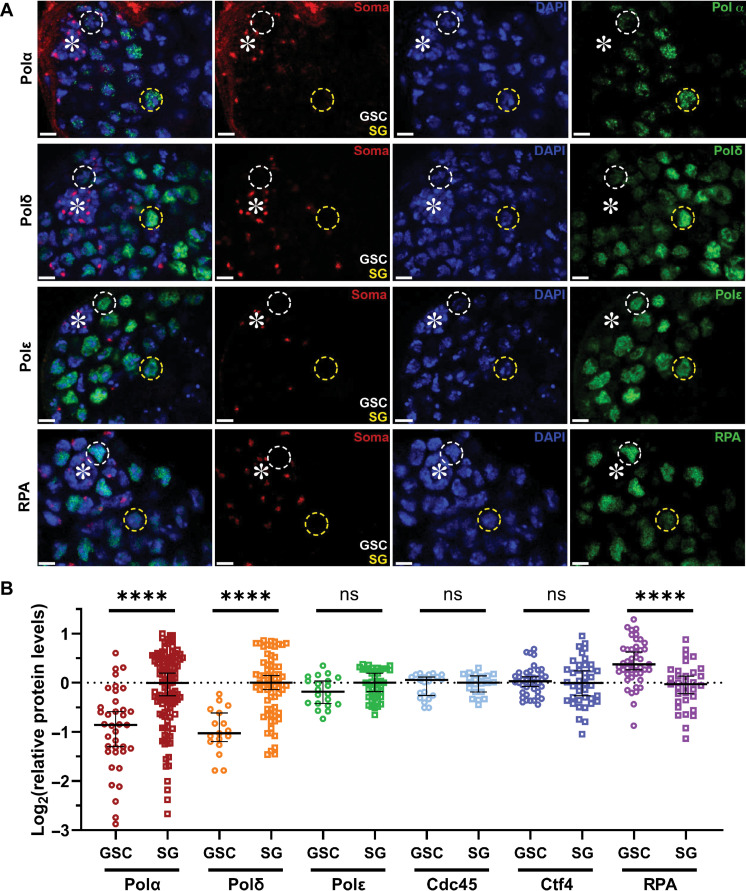
Distinct expression patterns of different replication machinery components in the *Drosophila* male GSC lineage. (**A**) Images of expression of 3× HA-tagged endogenous DNA polymerases Polδ, Polα, and Polε (see Materials and Methods), as well as the RPA70-EGFP expressed from a transgene with its own promoter (*49*). Somatic cell–enriched histone modification H4K20me2/3 (red) (*113*), 4′,6-diamidino-2-phenylindole (DAPI; blue), and the respective replication proteins (green). Both Polδ and Polα show decreased levels in GSCs, while Polε has comparable expression between GSCs and SGs. Contrastingly, RPA is more enriched in GSCs compared to SGs using a transgene under the control of its endogenous regulatory elements (*rpa70 > rpa70-EGFP*) (*49*). Representative GSCs are indicated by the white dotted circle, while SGs are indicated by the yellow dotted circle. Asterisk: hub. Scale bars, 10 μm. (**B**) Quantification of the relative expression levels of different replication proteins, using normalization to SGs followed by log_2_ transformation (Materials and Methods). Medians: GSC Polα log_2_ = −0.86 (*n* = 37), SG Polα log_2_ = 0.00 (*n* = 118); GSC Polδ log_2_ = −1.03 (*n* = 17), SG Polδ log_2_ = 0.00 (*n* = 70); GSC Polε log_2_ = −0.18 (*n* = 20), SG Polε log_2_ = 0.00 (*n* = 43); GSC Cdc45 log_2_ = 0.06 (*n* = 21), SG Cdc45 log_2_ = 0.00 (*n* = 21); GSC Ctf4 log_2_ = 0.03 (*n* = 40), SG Ctf4 log_2_ = 0.00 (*n* = 40); GSC RPA log_2_ = 0.38 (*n* = 45), SG RPA log_2_ = −0.03 (*n* = 33). See table S1 for details. All ratios: Median ± 95% confidence interval. Mann-Whitney test, ****P < 10^−4^; ns, not significant.

To identify which factors could be responsible for stem cell–specific asymmetric histone inheritance, we performed a candidate gene screen using a series of CRISPR-Cas9–mediated knock-in lines with the hemagglutinin (HA) tag at individual genes that encode distinct key replication machinery components. The levels of proteins involved in lagging strand synthesis, such as DNA polymerases α and δ (Polα and Polδ), differ significantly between GSCs and SGs, with GSCs having 51% the levels of Polδ and 58% the levels of Polα as compared to SGs (Fig. 1, A and B). In contrast, a key component for leading strand synthesis, DNA polymerase ε (Polε), exhibits comparable levels between GSCs and SGs (Fig. 1, A and B). On the other hand, the single-stranded DNA (ssDNA) binding protein replication protein-A 70 (RPA70), the largest subunit of the ssDNA-binding heterotrimeric complex (*47*, *48*), displays the opposite trend with 54% more RPA expressed in GSCs compared to SGs (Fig. 1, A and B), using the RPA70–enhanced green fluorescent protein (EGFP) fusion protein expressed under the endogenous regulatory elements of the *rpa70* gene (*16*, *49*). Notably, when comparing S phase GSCs and S phase SGs using a pulse of the thymidine analog 5-ethynyl-2′-deoxyuridine (EdU), Polα, Polδ, and RPA—but not Polε—shows significant differences between these two staged germ cells (fig. S1, A to E). Other replication machinery components, such as the replication fork progression protein cell division cycle 45 (Cdc45), show no significant difference between GSCs and SGs (Fig. 1B). Another component whose yeast homolog has been shown to have histone chaperoning activities (*25*), chromosome transmission fidelity 4 (Ctf4), also displays statistically indistinguishable levels between GSCs and SGs (Fig. 1B and fig. S1F).

While the substantially reduced levels of lagging strand polymerases in GSCs could lead to relatively asynchronous DNA strand syntheses and result in excessive ssDNA, we hypothesize that the higher levels of RPA in GSCs are responsible for coating and stabilizing ssDNA to prevent DNA damage (*50*). Moreover, RPA is capable of competing with Polα at ssDNA sites, therefore preventing Polα from binding to and acting on the lagging strand (*51*–*55*). Therefore, decreased Polα and increased RPA could cooperatively contribute to measured lagging strand synthesis in GSCs. It is also plausible that this underlies the longer cell cycle length of GSCs than SGs, which has been reported previously (*56*).

Together, these results demonstrate differential expression of the lagging strand-enriched replication components in the *Drosophila* male germ line, with increased Polα and Polδ but reduced RPA70 in the more differentiated SGs compared to GSCs.

### Reducing Polα levels or inhibiting Polα activities increases old versus new histone separation in S phase nuclei of progenitor cells

**Fig. 2.**
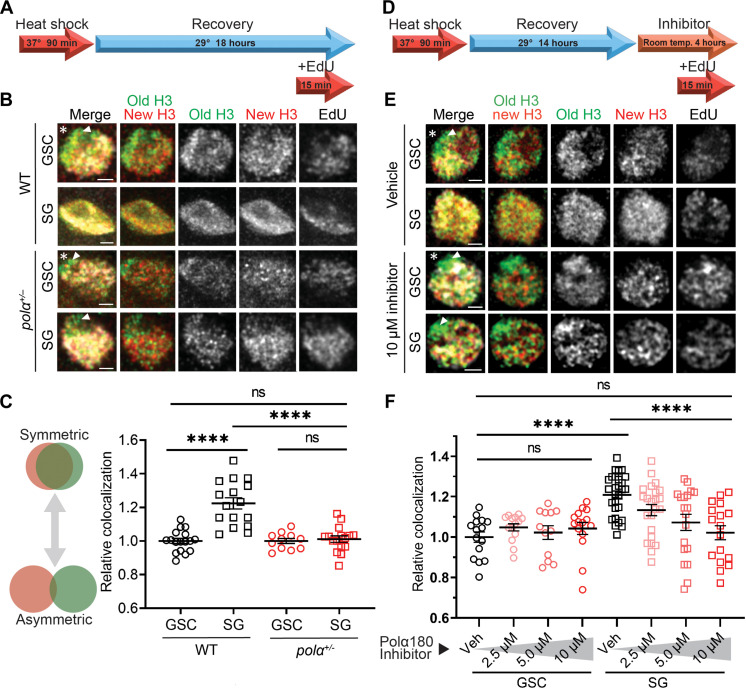
Compromising Polα activity increases old histone versus new histone separation in S phase nuclei of progenitor cells. (**A**) Regime testing old (EGFP) versus new (mCherry) histone localization pattern following heat shock–induced tag switch. (**B**) Airyscan images of representative control WT GSC, WT SG, *pol*α*50^+/−^* GSC, and *pol*α*50^+/−^* SG, respectively, in S phase nuclei wherein nucleoplasmic histones are largely washed off using a stringent clearance buffer. (**C**) Quantification of the correlation between old H3 and new H3 signals in S phase nuclei: WT GSC = 1.00 ± 0.06 (*n* = 17), WT SG = 1.22 ± 0.03 (*n* = 16), *pol*α*50^+/−^* GSC = 1.00 ± 0.02 (*n* = 11), *pol*α*50^+/−^* SG = 1.01 ± 0.02 (*n* = 16). See table S6 for details. (**D**) Regime testing old versus new histone localization pattern in response to Polα180 (or PolA1) inhibitor. (**E**) Airyscan images of representative GSCs and SGs treated with vehicle or Polα180 inhibitor for 4 hours before clearance buffer treatment and fixation. Arrowheads in (B) and (E): Unreplicated regions are enriched with old H3 but depleted with new H3 and EdU labeling. (**F**) Quantification of the correlation between old H3 and new H3 signals in S phase nuclei following inhibitor treatment: Vehicle GSC = 1.00 ± 0.03 (*n* = 15), 2.5 μM GSC = 1.05 ± 0.02 (*n* = 14), 5.0 μM GSC = 1.02 ± 0.03 (*n* = 12), 10 μM GSC = 1.04 ± 0.03 (*n* = 15); vehicle SG = 1.21 ± 0.02 (*n* = 28), 2.5 μM SG = 1.13 ± 0.03 (*n* = 23), 5.0 μM SG = 1.07 ± 0.04 (*n* = 19), 10 μM SG = 1.02 ± 0.04 (*n* = 17). See table S7 for details. In all merged images: old H3 (green), new H3 (red), as well as EdU (white), Arm (not shown but used as hub marker). Asterisk: hub. Scale bars, 1 μm. All images and quantifications for SGs use four-cell SGs. All ratios: Mean ± SEM. Mann-Whitney test, ****P < 10^−4^.

On the basis of the above observation, we hypothesize that potentially asynchronized strand synthesis could bias old histone recycling to the leading strand at individual replication forks, serving as a key molecular mechanism underlying asymmetric histone incorporation in GSCs. To investigate this hypothesis, we first examined the distribution of old versus new histones in intact nuclei using a dual-color system to label old H3 with EGFP and new H3 with mCherry in the male germ line (*16*, *22*). Using a heat shock–induced tag switch (Materials and Methods), canonical new histones are mainly incorporated during S phase, as shown previously in the male germ line (*15*, *57*), as well as in other adult stem cell lineages (*17*, *18*). To avoid any possible complications caused by nonchromatin-bound histones, we used a stringent clearance buffer that has been shown to effectively remove free histones in the nucleus (*22*, *58*, *59*). This strategy, together with Airyscan microscopy (*16*, *20*, *60*), with a spatial resolution of approximately 120 to 150 nm, allows us to visualize separable old H3-EGFP– versus new H3-mCherry–enriched regions in the control wild-type (WT) GSCs at S phase, labeled with a pulse of EdU (Fig. 2, A and B). Using these assays, the degree of separation between old and new H3 is less in WT SGs than that in WT GSCs during S phase (Fig. 2B). Quantification using a relative Pearson colocalization measurement (*17*, *18*, *57*) reveals a significantly higher degree of colocalization between old H3-EGFP and new H3-mCherry in WT SGs than in WT GSCs (Fig. 2C), consistent with more symmetric incorporation of old versus new histones in S phase WT SGs compared to WT GSCs.

We next asked whether compromising lagging strand synthesis in SGs could recapitulate GSC-like features, such as separable old versus new histone patterns in the S phase nuclei. Since replication components are essential for animal survival and cell cycle progression, we sought to compromise lagging strand polymerases without causing severe phenotypes. The *pol*α*50* gene (Materials and Methods) encodes the DNA primase subunit 1 (or Prim1). The null *pol*α*50* mutant is 100% homozygous lethal. Germ cells with strong *pol*α*50* loss-of-function condition using germline-specific RNA interference (RNAi) knockdown yield substantial cell death (fig. S2E), indicated by excess ssDNA and abnormal nuclear morphology (*61*), and this effect is stronger in SGs than GSCs. The *pol*α*50^+/−^* heterozygous flies are viable with no detectable phenotypes. Upon measuring the Polα180 protein levels in *pol*α*50^+/−^* germ cells, we observed the same relative levels of Polα180 in *pol*α*50^+/−^* GSCs versus SGs (fig. S2A), as seen in the WT germ line (Fig. 1B), suggesting that decreasing one catalytic lagging strand replication component does not disrupt the expression patterns of another. In addition, measurement of S phase indices, representing the percentage of cells in the S phase, shows no significant differences between WT and *pol*α*50^+/−^* GSCs or SGs (fig. S2B), suggesting that S phase progression of germ cells is not impaired.

Because Polα50 is an essential replication component, we also tested cellular and genome stability by measuring cell death, as reported by a LysoTracker (*62*, *63*), and phosphorylated H2Av (γH2Av) staining (*64*, *65*), respectively. We found that both the LysoTracker-labeled germline cysts and the quantified γH2Av immunostaining signals show no significant differences between WT and *pol*α*50^+/−^* testes, suggesting that germ cells cooperate well with reduced levels of Polα50 (fig. S2, C and D). This may partly result from the germline-specific increase in RPA levels, as observed in comparisons with cyst stem cells in the testis (fig. S1, D and E). Sufficient RPA may protect ssDNA generated by lagging strand synthesis that is decoupled from the opening of the replication fork. When the primase levels are reduced in *pol*α*50^+/−^* males, S phase SGs exhibit markedly more separable patterns between old and new H3, comparable to those detected in WT GSCs and *pol*α*50^+/−^* GSCs during S phase (Fig. 2, B and C).

In addition to this genetic approach, we tried a pharmacological strategy with a Polα inhibitor that prevents the DNA binding ability and primer elongation activity of DNA polymerase α subunit 1 (PolA1 or Polα180; fig. S3A) (*66*, *67*). At a high concentration (e.g., 100 μM), this inhibitor completely blocks DNA replication, indicated by the absence of EdU incorporation in different staged germ cells (fig. S3C). However, when using it at a relatively low concentration (e.g., 10 μM), normal DNA replication could proceed with proper EdU incorporation compared to the control sample treated with vehicle (Fig. 2D and fig. S3, B and C). With this inhibitor treatment, SGs also exhibit separable old versus new H3 patterns similar to those detected in the *pol*α*50^+/−^* cells (inhibitor SG in Fig. 2E versus *pol*α*50^+/−^* SG in Fig. 2B). The inhibitor-treated SGs show more separation than the vehicle-treated SGs and display patterns comparable to either inhibitor-treated GSCs or vehicle-treated GSCs (Fig. 2E). Quantifications further reveal that this inhibitor induces old versus new H3 separation in SGs in a dosage-dependent manner (Fig. 2F). Contrastingly, it causes no significant changes in GSCs regardless of the concentration (Fig. 2F). Notably, the presence of intermediate histone separation patterns at decreasing concentrations of inhibitor (e.g., 5.0 and 2.5 μM) indicates that the asymmetric histone incorporation pattern is tunable and scales to the inhibition of Polα. In addition, because GSCs are relatively unaffected, we hypothesize that these cells are at the maximum of histone asymmetry and thus cannot be made more asymmetric by inhibiting Polα activity.

Moreover, in all imaged nuclei undergoing DNA synthesis, unreplicated regions are enriched with old H3 but devoid of new H3 as well as EdU labeling (arrowheads in Fig. 2, B and E). Here, we found that vehicle-treated GSCs exhibit higher colocalization of EdU with new histones, likely due to the separate incorporation of old and new histones in control GSCs (fig. S3D). Meanwhile, vehicle-treated SGs show no such bias in EdU colocalization, likely because old and new histones are incorporated together in control SGs (fig. S3D). However, upon treatment with the Polα inhibitor, SGs exhibit greater colocalization of EdU with new H3, resembling the pattern observed in GSCs (fig. S3D). To confirm that the EdU bias did not influence our old-new H3 colocalization results, we quantified histone colocalization strictly within the EdU-positive regions of each nucleus (fig. S3E). The effect of the Polα inhibitor in inducing greater separation of old and new H3 in SGs persists even when analysis is restricted to actively replicating regions (fig. S3F). In summary, these data in intact S phase nuclei demonstrate that reducing primase levels or inhibiting Polα activity is each sufficient to induce separable old versus new histone incorporation in S phase SGs, to a degree indistinguishable from that in GSCs.

### Reducing Polα levels enhances asymmetric old histone incorporation at the replication fork in S phase progenitor cells

Next, to directly visualize the dynamic histone incorporation patterns at the actively replicating regions, a short pulse of EdU was introduced in combination with a single-molecule chromatin fiber technique (*16*, *20*). To precisely label chromatin fibers derived from GSCs versus SGs, we paired the Gal4 transcription activator controlled by the early germline-specific *nanos* driver (*nos-Gal4*Δ*VP16*) (*68*) with the Gal80 transcription repressor under the control of the *bag of marbles* promoter (*bam-Gal80*), which turns on expression from two-cell to late-stage SGs (*69*). This combination restricts the *H3-EGFP* transgene expression almost exclusively in GSCs with some detectable expression in the gonialblasts but almost undetectable signals in the SGs, which differs from the early-stage germ cell expression pattern driven solely by *nos-Gal4* (*70*) and late-stage germ cell expression pattern driven solely by *bam-Gal4* (fig. S4A) (*71*–*73*). These germline stage-specific expression patterns are confirmed by quantification using a *H3-EGFP* reporter (fig. S4B).

**Fig. 3.**
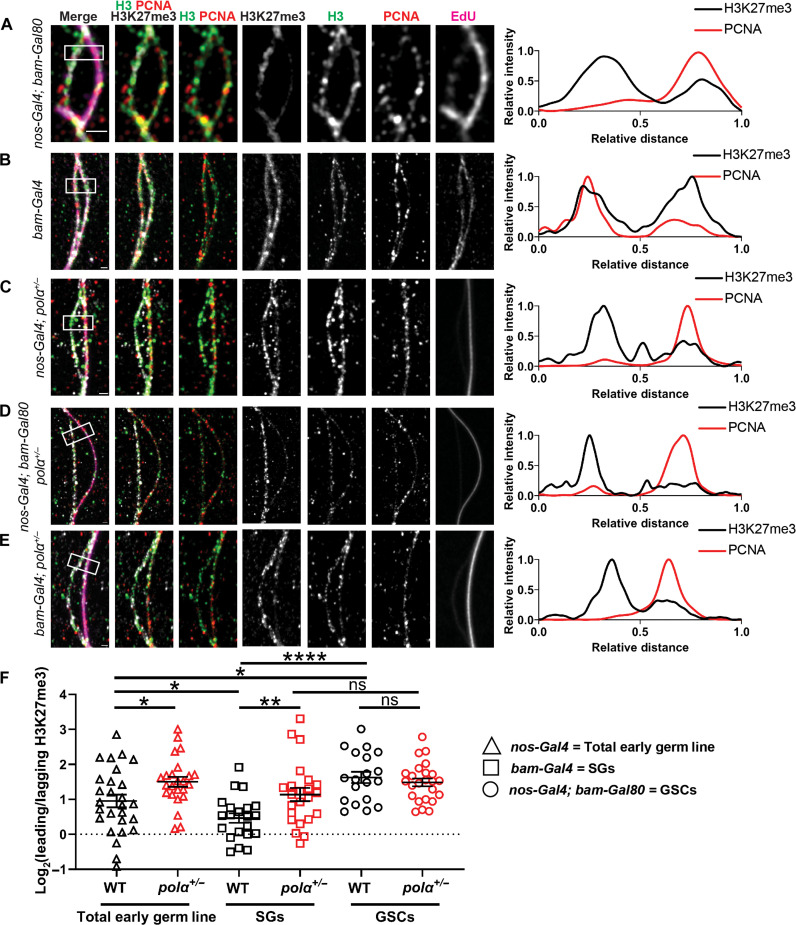
Reducing Polα levels enhances asymmetric old histone recycling at the replication fork in progenitor cells. (**A** to **E**) Airyscan images of chromatin fibers isolated from testes with the following genotypes: (A) *nos-Gal4*Δ*VP16; bam-Gal80 > H3-EGFP*, (B) *bam-Gal4 > H3-EGFP*, (C) *nanos-Gal4 > H3-EGFP;pol*α*50^+/−^*, (D) *nos-Gal4*Δ*VP16; bam-Gal80 > H3-EGFP;pol*α*50^+/−^*, (E) *bam-Gal4 > H3-EGFP;pol*α*50^+/−^*, respectively. In all merged images: H3K27me3 (white), H3-EGFP (green), PCNA (red), and EdU (magenta). All images are accompanied by a line plot showing the distance-dependent H3K27me3 and PCNA signals over the indicated region (white outlined box). Scale bars, 1 μm. (**F**) Quantification of the H3K27me3 signals on chromatin fibers in log_2_ scale: *nanos-Gal4 > H3-EGFP* = 0.95 ± 0.18 (*n* = 27), *nanos-Gal4 > H3-EGFP;pol*α*50^+/−^* = 1.50 ± 0.14 (*n* = 24), *bam-Gal4 > H3-EGFP* = 0.46 ± 0.14 (*n* = 21), *bam-Gal4 > H3-EGFP;pol*α*50^+/−^* = 1.14 ± 0.19 (*n* = 23), *nos-Gal4*Δ*VP16; bam-Gal80 > H3-EGFP = 1.63 ± 0.16* (*n* = 19), *nos-Gal4*Δ*VP16; bam-Gal80 > H3-EGFP;pol*α*50^+/−^* = 1.49 ± 0.11 (*n* = 24). See table S16 for details. All ratios: Mean ± SEM. Mann-Whitney test, ****P < 10^−4^, **P < 0.01, and *P < 0.05.

Using the *H3-EGFP* reporter with different drivers, we labeled chromatin fibers derived from early-stage germ cells including GSCs (*nos > H3-EGFP*), from very early-stage germ cells enriched with almost exclusive GSCs (*nos-Gal4*Δ*VP16; bam-Gal80* > *H3-EGFP*), and from late-stage SGs (*bam* > *H3-EGFP*). We then explored old histone recycling patterns at the H3-EGFP–labeled and EdU-positive chromatin fibers using the old H3-enriched H3K27me3 histone modification (*36*, *74*, *75*). We also distinguished the strandedness with the lagging strand-enriched component proliferating cell nuclear antigen (PCNA) (*16*, *76*). Together, chromatin fibers carrying all four markers (i.e., H3-EGFP, EdU, anti-H3K27me3, and anti-PCNA) were analyzed using Airyscan microscopy. While there are technical limitations to this method (see Materials and Methods), careful selection for clear replication bubbles with these markers captures the histone incorporation patterns in recently replicated stretches of DNA. In particular, nearby stretches of DNA are quantified to infer specific sister loci that were replicated by the passage of one fork. Symmetric forks would result in these sampled loci demonstrating relatively similar levels of parental histone incorporation, while asymmetric forks would result in loci that have differing parental histone incorporation. While *nos > H3-EGFP*–labeled chromatin fibers show a relatively wide distribution of H3K27me3 between replicative sister chromatids (fig. S4, C and D) with an overall biased distribution toward the PCNA-depleted leading strand (Fig. 3F and fig. S4E), *nos-Gal4*Δ*VP16; bam-Gal80* > *H3-EGFP*–labeled chromatin fibers show consistently more asymmetric H3K27me3 distribution toward the leading strand (Fig. 3, A and F, and fig. S4E). In contrast, *bam* > *H3-eGFP*–labeled fibers display a more symmetric H3K27me3 distribution pattern (Fig. 3, B and F, and fig. S4E). Notably, previous reports using an imaging-based proximity ligation assay in intact nuclei (*77*, *78*) demonstrate higher proximity of new histones to lagging strand-enriched Ligase or PCNA in GSCs but not in SGs (*16*), consistent with the results shown here.

To quantify old histone incorporation patterns, we used H3K27me3 as a proxy for old histones and plotted its ratio on the PCNA-depleted leading strand to the PCNA-enriched lagging strand [log_2_ ratios in Fig. 3F and fig. S4 (E and I)]. The *nos-Gal4*Δ*VP16; bam-Gal80*–labeled and the *bam*-labeled chromatin fibers are not only statistically distinguishable from each other (*P* < 10^−4^, Fig. 3F) but also statistically different from the *nos*-labeled group (*P* < 0.05, Fig. 3F). Combining the *nos-Gal4*Δ*VP16; bam-Gal80*–labeled and *bam*-labeled groups in silico generates a dataset indistinguishable from the *nos*-labeled group (fig. S4E), suggesting that the heterogeneity of both GSC-derived and SG-derived fibers could underlie the detected H3K27me3 variation among the *nos*-labeled chromatin fibers.

Furthermore, we found that the *nos*-labeled fibers from *Pol*α*50^+/−^* testes show more asymmetric H3K27me3 distribution toward the leading strand than the *nos*-labeled fibers from the control (Fig. 3, C and F). Consistently, the *nos*-labeled fibers from heterozygotes of the *pol*α*180* gene, which encodes DNA polymerase α subunit 1 (or PolA1), also exhibit a more asymmetric H3K27me3 distribution pattern toward the leading strand than those from the control (fig. S4, I and J). In contrast, compromising Polα50 has little effect on *nos-Gal4*Δ*VP16; bam-Gal80*–labeled chromatin fibers (Fig. 3, D and F). Notably, *bam > H3-eGFP*–labeled chromatin fibers display significantly more asymmetric patterns in the *pol*α*50^+/−^* samples than in the control (Fig. 3, E and F), in accordance with the results shown in intact S phase nuclei (Fig. 2, B and C). This result holds for *bam > H3-eGFP*–labeled fibers from control tissue after treating with the Polα inhibitor (fig. S4, F to H), indicating that both genetic and pharmacological manipulation of lagging strand replication have similar effects at the replication fork.

Together, these results demonstrate that compromising Polα affects SGs with normally high levels of Polα (i.e., *bam*-labeled chromatin fibers in Fig. 3 and intact SG nuclei in Fig. 2) more than GSCs that already have low levels of Polα (i.e., *nos-Gal4*Δ*VP16; bam-Gal80*–labeled chromatin fibers in Fig. 3 and intact GSC nuclei in Fig. 2). It is likely that reducing Polα levels below a certain threshold cannot further increase histone asymmetry, but reducing Polα from relatively high levels (i.e., SG-like) to relatively low levels (i.e., GSC-like) is sufficient to enhance asymmetric old histone recycling at the replication fork.

Last, to test whether RPA also contributes to asymmetric histone incorporation, we overexpressed the *rpa70* cDNA using *nos-Gal4* (*nos > rpa70-HA*). Likewise, the overexpression of RPA70 results in enhanced asymmetric H3K27me3 incorporation at the replicative regions, indicating that increased levels of RPA lead to enhanced asymmetric old histone recycling (fig. S4, I and K). Notably, these results are consistent with the previous report that using the *rpa-70 > rpa-70-EGFP* line, where the transgenic RPA-70-EGFP fusion protein is under the control of the endogenous *rpa-70* regulatory elements and represents a slight overexpression condition. Under this condition, an average of 3.2-fold leading strand-biased H3K27me3 asymmetry is detected, more than the control line which shows an average of 2.0-fold leading strand-biased H3K27me3 asymmetry (*16*). These effects could be attributed to the previously reported competing roles of RPA in preventing Polα from binding to the lagging strand (*51*–*55*). In summary, the chromatin fiber results demonstrate that SGs with relatively high levels of Polα and low levels of RPA can be induced to have GSC-like asymmetric old histone incorporation at the replicative regions by reducing Polα levels or by enhancing RPA expression.

### Reducing Polα levels induces differential condensation of old histone- versus new histone-enriched regions in M phase progenitor cells

**Fig. 4.**
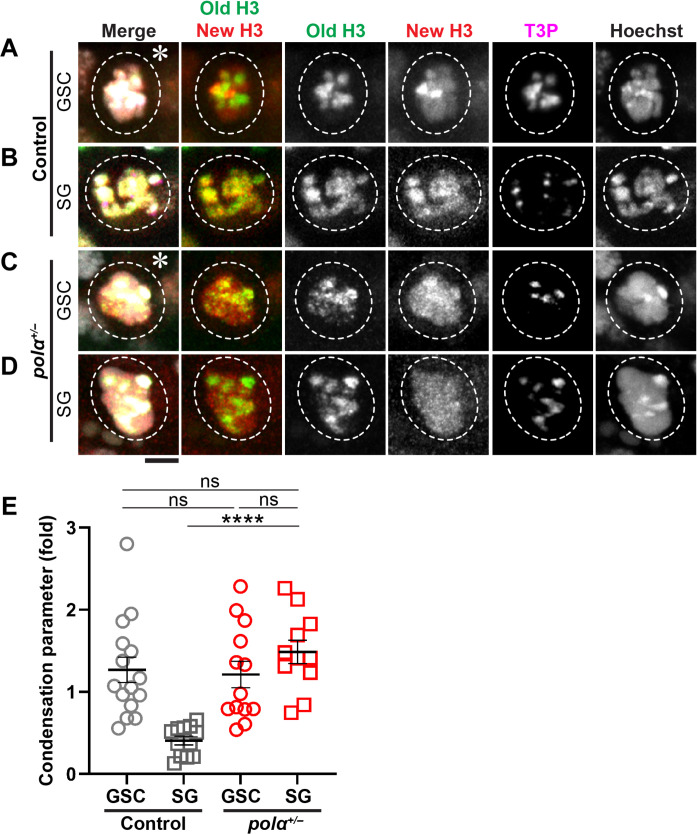
Reducing Polα levels induces differential condensation of old H3- versus new H3-enriched regions in M phase progenitor cells. (**A** and **B**) Representative images of: (A) an M phase GSC showing more compact old H3-enriched regions than new H3-enriched regions, positive with a mitotic marker anti-H3T3ph, H3T3P, or T3P (*114*); (B) an M phase eight-cell SG showing equally compact old H3-enriched and new H3-enriched regions (positive with T3P) in the control WT testes. (**C** and **D**) Representative images of: (C) an M phase GSC and (D) an M phase eight-cell SG in the *pol*α*50^+/−^* testes, both showing more compact old H3-enriched regions than new H3-enriched regions, positive with a mitotic marker anti-T3P (*114*). (**E**) Compaction index in log_2_ scale: Control GSC = 1.27 ± 0.15 (*n* = 15), control eight-cell SG = 0.41 ± 0.05 (*n* = 12), *pol*α*50^+/−^* GSC = 1.21 ± 0.16 (*n* = 13), and *pol*α*50^+/−^* eight-cell SG = 1.48 ± 0.14 (*n* = 11). See table S17 for details. The control compaction index data are from (*22*) with permission. All ratios: Mean ± SEM. Mann-Whitney test, ****P < 10^−4^.

It has been reported that old H3- versus new H3-enriched chromosomal regions display differential condensation in the M phase GSCs but overlapping patterns in the M phase SGs (*22*). Consistent with previous reports (*22*, *59*), the control GSCs and SGs display marked condensation differences between old H3- and new H3-enriched regions (Fig. 4, A and B), while the *pol*α*50^+/−^* SGs (Fig. 4D) show GSC-like (Fig. 4, A and C) differential condensation patterns. Here, using a relative chromatin compaction index to measure the differential condensation between old H3- and new H3-enriched regions as reported previously (*22*, *59*), a significant difference could be detected between GSCs and SGs in the control testes but not between GSCs and SGs in the *pol*α*50^+/−^* testes (Fig. 4E). In the *pol*α*50^+/−^* testes, both GSCs and SGs display similar patterns compared to the control GSCs but notably distinct patterns compared to the control SGs (Fig. 4E). Collectively, these results demonstrate that by compromising a single lagging strand-enriched component, differential condensation of old H3- versus new H3-enriched regions in M phase cells, a GSC-specific feature, can be recapitulated in the SGs.

### Detectable asynchrony between leading strand and lagging strand syntheses

**Fig. 5.**
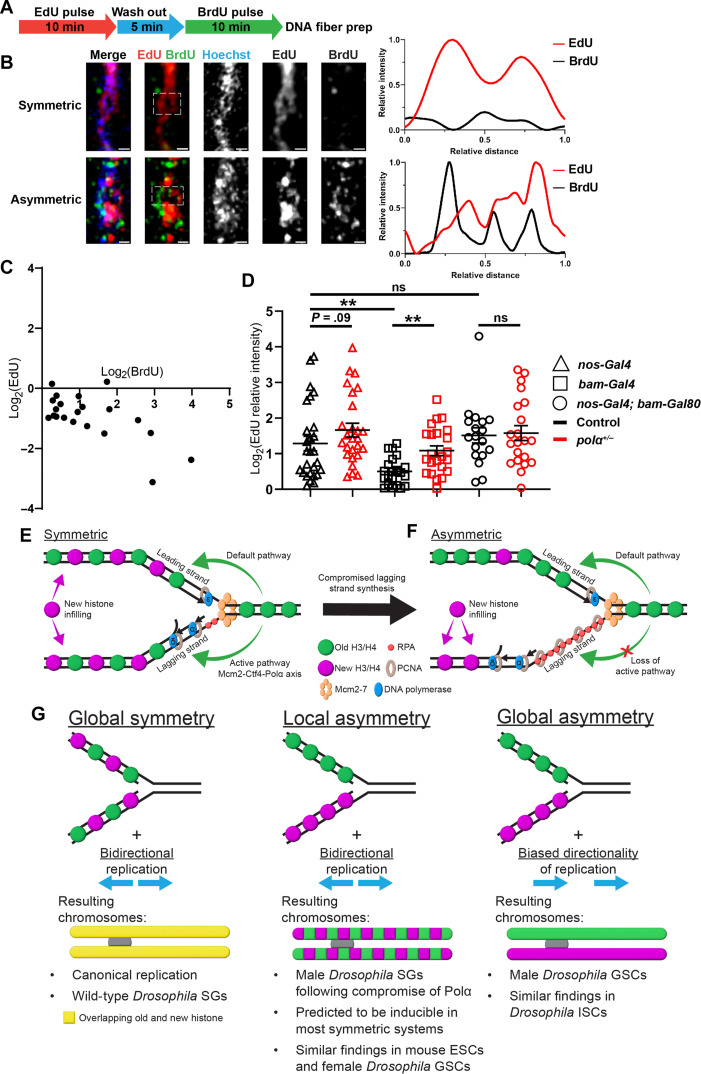
Asynchronous leading strand versus lagging strand syntheses. (**A**) Regime with a 10-min EdU pulse followed by a 5-min wash out and then a 10-min BrdU pulse to label DNA fibers (Materials and Methods). (**B**) Airyscan images of DNA fibers: The two line plots correspond to a representative symmetric replicative region with EdU labeling both strands (white dotted outline, top) and an asymmetric region where EdU and BrdU are on opposing strands (white dotted outline, bottom). Scale bars, 1 μm. (**C**) Log_2_-scale two-dimensional (2D) plot showing the distribution of BrdU and EdU on the DNA fibers containing both signals. Most fibers display EdU and BrdU on the opposing DNA strands. See table S22 for details. (**D**) Quantification of EdU distribution in log_2_ scale, introduced by a 15-min EdU pulse, on chromatin fibers labeled with H3-EGFP driven by the following drivers without strandedness information: *nanos-Gal4* = 1.28 ± 0.21 (*n* = 27), *nanos-Gal4;pol*α*50^+/−^* = 1.66 ± 0.19 (*n* = 26), *bam-Gal4* = 0.50 ± 0.09 (*n* = 21), *bam-Gal4;pol*α*50^+/−^* = 1.08 ± 0.14 (*n* = 23), *nos-Gal4*Δ*VP16; bam-Gal80* = 1.51 ± 0.21 (*n* = 18), *nos-Gal4*Δ*VP16; bam-Gal80;pol*α*50^+/−^* = 1.58 ± 0.21 (*n* = 21). See table S23 for details. All ratios: Mean ± SEM. Mann-Whitney test, **P < 0.01. (**E** and **F**) Models depicting incorporation at the replication fork: (E) In symmetrically dividing cells, comparable leading strand versus lagging strand syntheses result in equal histone recycling to either strand, based on previous reports (*24*, *25*, *29*). (F) In asymmetrically dividing cells, reduced levels of lagging strand polymerases lead to delayed lagging strand synthesis relative to the leading strand, which biases old histone recycling to the leading strand. (**G**) Local histone asymmetry at replication forks plus biased replication direction results in global asymmetry (last column). Asymmetry at forks alone results in local domains of asymmetry only along chromosomes (middle column).

Next, to measure the leading versus lagging strand syntheses in the early-stage germ line, we attempted to directly visualize these processes using active incorporation of nucleotide analogs. Previously, it has been shown that the syntheses of the two DNA strands can be discontinuous where the leading and lagging strand polymerases are not tightly coupled in *Escherichia coli* (*79*) or when applying a PolA1 inhibitor in cultured human cells (*80*). To investigate whether this asynchrony exists and is detectable in the *Drosophila* testes, we investigated whether asynchronous DNA strand syntheses can be differentially labeled qualitatively, using distinct nucleotide analogs introduced in a sequential order [e.g., a short pulse of EdU followed by a short pulse of bromodeoxyuridine (BrdU); Fig. 5A]. Using this regime, DNA fibers where both strands are colabeled with just one nucleotide (e.g., EdU) should represent regions where both strands are replicated within the time window of the EdU pulse (top of Fig. 5B). However, DNA fibers with EdU and BrdU on opposing strands likely capture the uncoupled syntheses of the two strands (bottom of Fig. 5B). The DNA fibers derived from the apical testis tips display the latter pattern in approximately 40% of the fibers (fig. S5A). On average, DNA fibers carrying both EdU and BrdU display a 2.35-fold BrdU enrichment toward one strand whereas a 1.91-fold EdU enrichment toward the opposing strand (Fig. 5C and fig. S5B).

On the other hand, when a single short EdU pulse is introduced on early germline-derived chromatin fibers where strandedness can be determined, 52% of them display a strong bias (≥2-fold) toward one of the two strands, with 79% of them displaying strong asymmetry toward the lagging strand (fig. S5, C and D), likely due to lingering lagging strand synthesis and thus a higher opportunity to incorporate EdU (fig. S5E). Consistently, PCNA signals often display asymmetric distribution on early germline-derived chromatin fibers, along with EdU, toward the H3K27me3-depleted lagging strand (Fig. 3A and figs. S4C and S5D).

To further test whether asynchronous DNA strand syntheses could be mechanistically responsible for asymmetric histone incorporation, we analyzed the EdU signals of all chromatin fibers that have been analyzed for the H3K27me3 patterns in Fig. 3. Very early-stage germline-derived (*nanos-Gal4*Δ*VP16; bam-Gal80*) chromatin fibers show a high degree of asymmetric EdU patterns, while the late-stage germline-derived (*bam-Gal4*) chromatin fibers primarily show symmetric EdU distribution between sister chromatids (Fig. 5D and fig. S5F). Notably, EdU asymmetry is substantially enhanced in the late-stage germline-derived (*bam-Gal4*) chromatin fibers from the *pol*α*50^+/−^* testes than that from the control (*P* < 0.01 in Fig. 5D and fig. S5F). The small incidence of leading strand- but a large population of lagging strand-biased EdU incorporation (fig. S5, D and F) are consistent with the hypothesis that there is temporal asynchrony between leading strand and lagging strand syntheses, with the lagging side taking more time on average (fig. S5E). For this assay, we measured the probabilities of the pulsed EdU to be incorporated on each strand qualitatively. The strand whose replication lingers has a relatively higher probability to incorporate EdU than the strand which does not. Overall, these results show that compromising Polα is sufficient to increase asymmetric H3K27me3 incorporation by the leading strand, likely by enhancing the temporal differences between sister chromatid syntheses.

Last, to directly visualize potentially asynchronous DNA strand syntheses, we used an endogenously tagged *cdc45* gene, resulting in the Cdc45-mCherry fusion protein as a marker for the Cdc45-MCM-GINS complexes (*81*, *82*), to label actively progressing replication forks. Consistent with its role, chromatin fibers labeled with Cdc45 and a short pulse of EdU show that Cdc45 localizes at the leading edge of the EdU label (fig. S5, G and H). We also observed that germline-derived fibers show more unidirectionality on consecutive forks than somatic cell–derived fibers (fig. S5I), which was previously reported using an alternative approach (*16*). We then performed immunostaining using antibodies against the HA tag to label Polα-HA for lagging strand polymerase and Polε-HA for leading strand polymerase, using the endogenously tagged genes, respectively. While Polε is always tightly associated with Cdc45 at EdU-labeled replicative chromatin fibers (fig. S5, J and L), Polα could be found in tracts extending away from Cdc45 (fig. S5, K and L), indicating that Polα is spatially decoupled from the actively progressing fork. Together, these results demonstrate that the temporal and spatial separation between leading strand and lagging strand syntheses can be visualized by nucleotide analogs introduced at different time points during DNA replication or with different replisome components.

## DISCUSSION

Here, we report a crucial molecular mechanism underlying asymmetric histone incorporation in stem cells. In symmetrically dividing progenitor cells, synchronized leading versus lagging strand syntheses give the old histone equal opportunities to be recycled by both strands (Fig. 5E), as shown previously (*24*, *25*, *29*, *31*, *36*). In asymmetrically dividing GSCs, reduced lagging strand polymerase levels could suppress lagging strand synthesis relative to the leading strand, which results in a pronounced temporal difference. This difference could bias displaced old histone ahead of the fork to be immediately recycled by the leading strand, whereas new histones infill to the lagging strand (Fig. 5F). This model is consistent with a previous report that nucleosomes have the priority to be reincorporated by the double-stranded leading strand in vitro (*83*). The increased expression of RPA in stem cells could also facilitate this process (Fig. 5F). This model is also consistent with the previous imaging-based results displaying abundant RPA bound to the lagging strand on early-stage germ cell–derived chromatin fibers (*16*). Either reducing the expression of the key lagging strand polymerase or inhibiting its activity is sufficient to induce stem cell–specific asymmetric histone incorporation patterns even in nonstem progenitor cells.

Notably, although SGs with approximately 50% primase levels in *pol*α*50* heterozygotes have S phase–specific replication-dependent histone incorporation patterns (Figs. 2 and 3) and M phase–specific differential chromosomal condensation patterns (Fig. 4), similar to those in GSCs, these patterns could reflect “local” asymmetry instead of “global” asymmetry as detected in asymmetric division of male GSCs (*15*, *16*). However, SGs do not reside in a polαrized microenvironment like the “niche” for GSCs. In addition, there is no evidence that the microtubule organization centers, the centrosomes, have asymemtric activities in WT SGs (*72*, *84*), whereas asymmetric centrosomes allow asymmetric mitotic machinery activity in GSCs (*21*). Therefore, these local chromatin asymmetries may not result in substantial differences between the two daughter cells resulting from SG symmetric cell division, unlike the asymmetric division of GSCs. Last, the *pol*α*50^+/−^* SGs seem to undergo terminal differentiation properly, as there are no obvious germline defects detectable in the *pol*α*50^+/−^* males. It is plausible that the molecular features such as the transcriptome and proteome of the *pol*α*50^+/−^* SGs remain unchanged or have inconsequential changes, despite the detectable changes of their chromatin structure. This indicates that the epigenome potentiates cell fate change but may not be determinstic for such a decision. The 50% reduction of primase in heterozygous SGs is analogous to the protein level change detected in WT GSCs compared to WT SGs (Fig. 1B).

Furthermore, we focus on the Polα-primase complex in this study because it predominantly acts on the lagging strand except the initial priming event on the leading strand and during rare repriming events at stalled replication forks (*52*, *85*, *86*). On the other hand, Polδ could contribute to the synthesis of both the leading strand and the lagging strand (*87*, *88*). Although it is known that Polα (*25*, *31*) and RPA (*89*) also play a role in chaperoning histones during replication-coupled nucleosome assembly, such activities have been demonstrated using specific mutations at their histone interacting domains. Investigating their chaperoning activities in regulating the male germ line would be intriguing, especially with a carefully designed experiment inducing germ cells carrying these mutations in a controllable manner (*90*). It would also be interesting to study other DNA replication components with histone chaperone functions, such as yeast Dpb3 and Dpb4 (mammalian PolE3 and PolE4, respectively) involved in recycling old histones toward the leading strand (*24*, *31*, *91*). Now, it remains unclear of their functional fly homologs, given the highly diverged histone binding motifs and uncharacterized histone chaperoning biochemical activities of the *Drosophila* CHRAC-14 (putative PolE3 ortholog) (*92*, *93*) and PolE4. Recently, a histone chaperone that coordinates old histone recycling to both leading and lagging strands has been reported (*94*–*96*), further demonstrating the complexity of this process. Here, our studies make use of genetic approaches that either compromise function or change expression of the full-length proteins. In addition, the pharmacological method uses inhibitors whose effects are more related to their enzymatic roles as replication machinery components, such as primer elongation, rather than chaperones. Notably, DNA replication inhibitors are often used to target overproliferative cancer cells. The drug adarotene and its derivative molecule used in this study have been shown to have anticancer properties in mice (*66*, *97*, *98*). However, the dose used in our studies is much lower than the dose used for cancer therapy to ensure minimal effect on S phase progression (fig. S3, B to F). Therefore, both genetic and pharmacological approaches emphasize the importance of the control for the optimal Polα level or inhibitor dosage, which needs to be calibrated empirically in different systems.

Last, DNA replication is fundamentally an inherently asymmetric process wherein the synthesizing processes of the leading strand versus the lagging strand are widely divergent. Previous studies have shown examples of uncoupled leading strand versus lagging strand syntheses in bacteria and cultured cells (*79*, *80*, *99*, *100*), particularly in cases where Polα or its priming activity is compromised. It has long been recognized that the leading strand versus the lagging strand may have the potential to differentially incorporate nucleosomes (*101*). The old histone-enriched H3K9me3 has been shown to be recycled by the leading strand at the retrotransposon elements to repress their ectopic transcription in S phase mouse embryonic stem cells (*102*). Furthermore, it has been shown that DNA replication speed and timing underlie cell fate regulation in mammalian cells, including mouse and human cells (*103*–*106*). Here, our results indicate that the inherent asymmetry of DNA replication itself could be used to differentially regulate histone incorporation, and this process displays stage specificity within an endogenous adult stem cell lineage. These results point to a very exciting possibility that developmentally programmed expression of key DNA replication components could regulate the establishment of distinct epigenomes in a cell type– and stage-specific manner. Given that replication components as well as histone proteins and their respective modifications are highly conserved, exploring how this mechanism may be used in other developmental contexts across different multicellular organisms could be a very intriguing research direction (*6*, *107*). These elegant and efficient mechanisms could be used to balance differential versus equal epigenome establishment in asymmetrically versus symmetrically dividing cells, which could then affect plasticity versus fidelity in cell fate decisions during development, homeostasis, and tissue regeneration. Technically, although our current imaging-based methods cannot reveal sequence information, it does provide the cell type and stage specificities, which are very much needed to solve the difference between stem cells and nonstem cells within the same stem cell lineage in vivo. Together, our imaging-based work using an endogenous stem cell system complements with the state-of-the-art genomic studies in symmetrically dividing cells such as mouse embryonic stem cells (*29*–*35*) and unicellular organisms such as yeast (*24*–*28*). These findings contribute to a deeper understanding of histone dynamics during DNA replication across different cell types and organisms.

## MATERIALS AND METHODS

### Fly strains and husbandry

Fly strains were raised on standard Bloomington media. All flies were raised at 25°C unless noted otherwise. The following fly strains were used: *hs-flp* on the X chromosome (Bloomington Stock Center BL-26902), *nos-Gal4* (with *VP16*) on the second chromosome (*70*), *nos-Gal4* (without *VP16* or *DVP16*) on the second chromosome [from Y. Yamashita, Whitehead Institute, USA and used in (*68*)], *bam-Gal4* on the third chromosome (*73*), *bam-Gal80* on the third chromosome (from J. Mathieu and J.-R. Huynh, Collège de France, France), *UASp-FRT-H3-EGFP-FRT-H3-mCherry* on the second chromosome as reported previously (*16*), *pol*α*50* P-element insertion (BL-27205), *pol*α*180* P-element insertion (BL-31805), *pcna > EGFP-pcna* and *rpa > rpa-EGFP* [from E. Wieschaus, Princeton University, USA and used in (*49*)], and *Vasa-EGFP* and *Vasa-mApple* [from A. Nakamura] (*108*).

The *pol*α*50* P-element insertion (BL-27205) was verified by sequencing to be a null allele using the following primers: 5′-AGCTCCAATCGTGTATCTCTCT-3′ (specific to the 5′ untranslated region of the *pol*α*50* gene locus) and 5′-CAATCATATCGCTGTCTCACTC-3′ (specific to the P-element sequences of the EP insertion) were used to amplify the genomic sequences corresponding to the 5′ end of the *pol*α*50* gene locus, where the P-element insertion was located on the basis of the Flybase (https://flybase.org/, Flybase ID FBti0115580). Sequencing with this pair of primers confirmed that the P-element is inserted at a position nine base pairs downstream of the start codon, resulting in the coding sequence 5′-ATGCCCGAAcatgatgaaataacataa (lowercase sequences indicate the P-element insertion). This leads to eight codons followed by a stop codon (underlined). Hence, this allele results in an early stop codon that very likely represents a null loss-of-function allele of the *pol*α*50* gene. This allele is not homozygous viable and is maintained as a heterozygous stock over a balancer chromosome. All experiments using the *pol*α*50^+/−^* background were outcrossing the *pol*α*50*/*Balancer* stock to a WT stock to have the *pol*α*50* P-element insertion allele over a WT chromosome.

### Knockdown of *pol*α in the germ line of adult testes

The *UAS-pol*α*180 RNAi* flies were crossed with *nanos-Gal4* (without *VP16*)*; tub-Gal80^ts^* flies to generate *tub-Gal80^ts^*, *nos-Gal4 > pol*α*180 RNAi* male flies. Crosses were maintained at 18°C (permissive temperature for Gal80^ts^) to keep the Gal4 repressed; therefore no knockdown occurs during development. After eclosion, flies were shifted to the 29°C (restrictive temperature for Gal80^ts^) for 24 hours before dissection. Dissected testes were then placed in Schneider’s medium containing 20 μM EdU for 10 min. Samples were then fixed and immunostained using standard whole mount procedures.

### Generating knock-in fly strains

Endogenously tagged fly strains were generated by CRISPR-Cas9 with the genome editing service provided by Fungene Inc. (Beijing, China). The knock-in strains encoding the following proteins were generated and used in this study: Cdc45-mCherry (internally tagged between D163 and Q164), Cdc45-3×HA (internally tagged between D163 and Q164), DNA Polε 255-kDa subunit-3×HA (tagged at the C terminus), DNA polymerase α 180kD-3×HA (tagged at the C terminus), DNA polymerase δ-3×HA (tagged at the C terminus), and Ctf4-EGFP (tagged at the C terminus).

### Heat shock scheme

Flies with *UASp-FRT-H3-EGFP-FRT-H3-mCherry* along with any relevant genotypes were crossed with *hs-flp; nanos-Gal4* and raised at 25°C. Within 2 days of eclosure, adult male flies were transferred to a vial, and the vial was submerged underwater at 37°C for 90 min. Flies were then recovered at 29°C for 18 hours before dissection for experiments, with the exception of experiments using the PolA1 inhibitor, as described below.

### Whole mount immunostaining experiments

Immunostaining experiments were performed using standard procedure (*15*). Primary antibodies used were anti-Armadillo [Arm; 1:100; Developmental Studies Hybridoma Bank (DSHB), N2 7A1], anti–Traffic Jam (Tj; 1:100; from M. Van Doren, Johns Hopkins University, USA), anti–α-Spectrin (1:50; DSHB, 3A9), anti-γH2Av (1:1000; Rockland, 600-401-914), anti-PCNA (1:100; Santa Cruz, sc-56), anti-GFP (1:1000; Abcam, ab13970), anti-HA (1:200; Sigma-Aldrich, H3663), anti-mCherry (1:1000; Invitrogen, M11217), anti-H3K27me3 (1:400; Millipore, 07-449), anti-H4K20me2/3 (1:400; Abcam, ab78517), anti-H3S10ph (1:2000; Cell Signaling Technology, 9701), rabbit anti-H3T3ph (1:200; Millipore, 05-746R), anti-BrdU (1:200; Abcam, ab6326), and anti-ssDNA (1:100; DSHB, AB_10805144). BrdU analog was Invitrogen B23151 BrdU. Secondary antibodies were the Alexa Fluor–conjugated series (1:1000; Molecular Probes). Confocal images were taken on the Zeiss LSM800 (with Airyscan mode) with a 63× oil objective lenses or on the Leica SPE with 63× oil immersion lenses.

### Quantification of protein levels in the early germ line

Images were analyzed using the ImageJ software FIJI. Germline cyst stages were identified using an Arm signal to label the two cyst cells encapsulating each cyst. Average intensity values were recorded for the center Z-slice of each cell/nucleus of interest. For germ cells within one cyst, only one germline nucleus from the entire cyst was measured as one data point. For the comparison of protein levels of endogenously tagged proteins, immunostaining signals in GSCs and four-cell and eight-cell SGs were measured, and a background was subtracted using the postmitotic hub cells, which are devoid of signals from any of these replication components. Signal intensity from four-cell and eight-cell SGs was then normalized to the average intensity of GSCs from the same batch of testes. For the batch-based normalization, within one experimental batch, each data point is normalized to the average of WT GSCs in this corresponding batch. To compare data among different batches, the resulting values were then used to calculate the relative amount of GSC protein level to SG protein level (set to 1 to facilitate comparison) and plot on a log_2_ scale (Fig. 1B). The dataset shown in Fig. 1B is from germ cells at each corresponding differentiation stage. We also labeled S phase germ cells using a EdU pulse and quantified them separately. The results using S phase germ cells were similar to those using germ cells without distinguishing S phase from G_2_ phase (fig. S1, A to D). For cyst stem cell protein levels, measurements were taken from nuclei positive for Tj (*109*) and EdU adjacent to the hub.

For the comparison of the stage specificity of each driver or driver combination, *nanos-Gal4* by itself, *nos-Gal4*Δ*VP16; bam-Gal80* combination, or *bam-Gal4* by itself was crossed to the *UASp-FRT-H3-EGFP-FRT-H3-mCherry* transgene without *hs-flp*. The EGFP signals reflecting the relative strength of each driver or driver combination were quantified in the corresponding germline cyst stages, identified using Arm to label the two encapsulating cyst cells. The central slice of a representative nucleus was taken for each cyst measured as one data point. The cytoplasmic space was used as a background for subtraction. The EGFP signals were normalized to the stage with the highest relative signal intensity: For *nanos-Gal4* by itself, all quantifications were normalized to the signals in GSCs; for the *nos-Gal4*Δ*VP16; bam-Gal80* combination, all quantifications were also normalized to the signals in GSCs; for *bam-Gal4* by itself, all quantifications were normalized to the signals in the eight-cell SGs (fig. S4B).

### S phase colocalization imaging and analysis

To visualize potentially differential histone incorporation during S phase, we applied a clearance buffer which effectively removes nucleoplasmic protein as previously described (*22*, *58*). Briefly, the clearance buffer is prepared by mixing 989 μl of the clearance buffer stock solution [8.4 mM Hepes, 100 mM NaCl, 3 mM MgCl, 1 mM EGTA, 300 mM sucrose, 2% Triton X-100, and 2% bovine serum albumin (BSA) in double-distilled H_2_O (ddH_2_O)] with 1 μl of dithiothreitol (DTT) and 10 μl of protease inhibitor (100× Leupeptin). After dissection, tissue samples were incubated in 20 μM EdU (Invitrogen Click-iT EdU Imaging Kit, catalog no. C10340) for 15 min in Schneider’s media at room temperature. At the end of the 15 min, the Schneider’s media were drained, and the clearance buffer was added for 2 min at 4°C in darkness. Samples were then fixed in 4% paraformaldehyde (PFA), washed with 1× Phosphate Buffered Saline with 0.1% Triton X-100 (PBST), and then blocked in 3% BSA for 30 min. For robust signals, both the old H3-EGFP and new H3-mCherry were immunostained with antibodies (e.g., anti-EGFP and anti-mCherry) using standard procedures. The CLICK reaction was performed according to the manufacturer’s instructions to label EdU. The DNA dye Hoechst was also added at this step.

Images were acquired on the Zeiss LSM800 using Airyscan mode on a 63× oil immersion objective. All samples were imaged using the identical settings. GSCs were identified by their proximity to the hub region. When four-cell stage SGs were used, only one SG per cyst was analyzed to represent one data point. All images were analyzed using FIJI software. The Pearson score was recorded using the Coloc2 plugin for each nucleus, which was cropped to include just the nucleus as much as possible as delineated by the Hoechst signals. Colocalization values were taken in a pairwise manner for new H3/old H3, new H3/EdU, and old H3/EdU. For each batch of images, the average measurement of the control GSCs was set to 1, and the other treatments are normalized to control GSCs, to avoid batch variability. The resulting values are then used to calculatemean ± SEM for new H3/old H3 colocalization. To analyze new H3 and old H3 colocalization with EdU, the old H3/EdU Pearson score was subtracted from the new H3/EdU Pearson score to quantify whether EdU was more colocalized with new or old H3, and one resulting value per nucleus constituted one data point to calculate mean ± SEM.

To find old H3/new H3 colocalization in just the EdU-positive region of the nucleus, the built-in coloc mode on Imaris software was used. Individual nuclei were cropped in three-dimensions (3D) to isolate them, and then a channel mask was applied to only consider regions with EdU signal. Colocalization was quantified in 3D for all EdU-positive regions of the nucleus, and the Pearson score was recorded. Batches were recorded as described above, and the resulting values were then used to calculate mean ± SEM.

### Assessing testis integrity

To determine the S phase index of both GSCs and SGs, testes expressing endogenous Vasa-mApple were labeled with a 15-min pulse of EdU, and after fixation, they were stained for Arm and alpha-Spectrin. The total number of GSCs and four-cell SGs per tissue was counted without considering the EdU channel, and only after the total amount of each cell stage had been determined was the EdU channel turned on to assess the number of those cells in S phase. Each four-cell SG counted as one cyst, since all four nuclei are synchronous in the cell cycle.

To label dying germline cells, LysoTracker Red (Invitrogen, L7528) was applied to live dissected testes at 1:1000 concentration in Schneider’s medium for 30 min at room temperature, at which point they were immediately fixed. All LysoTracker-positive germline cysts were counted regardless of stage due to difficulty discriminating between stages in dying cells. The total number of labeled cysts was counted per tissue, and this number was one data point.

γH2Av levels were quantified in four-cell SGs in S phase to assess DNA damage. To quantify γH2Av levels after immunostaining, we used Imaris software. After identifying an EdU-positive four-cell SG, one nucleus was randomly selected and cropped in 3D to exclude other nearby nuclei. Once isolated, the Imaris surface identifier was used to define the outer bounds of the nucleus as defined by Hoechst signal. Once the nucleus was defined, the mean γH2Av level inside the nucleus was recorded. Values were normalized to the average value of the control.

### Inhibitor treatment and analysis

For S phase colocalization experiments using the inhibitor, flies were heat shocked as described above and left at 29°C to recover for 14 hours. Testes were then dissected and placed in incubation media for 4 hours, resulting in 18 total hours of postheat shock recovery. After incubation with the inhibitor at the designated concentrations, these tissues were processed for S phase colocalization analysis, described above, or chromatin fibers, described below.

Polα180 inhibitor (MedChemExpress, catalog no. HY-147812), a derivative of the classical inhibitor adarotene, was prepared in dimethyl sulfoxide (DMSO) as stock and stored at −20°C (for short term) and −80°C (for long term) according to the manufacturer’s instructions. Drug incubation was performed on testes in “live cell media” containing Schneider’s insect medium with insulin (200 μg/ml), 15% fetal bovine serum by volume, and 0.6× penicillin/streptomycin (*21*). Before experiments, incubation media was prepared by diluting inhibitor solution (or DMSO vehicle) to the proper concentration in live cell media. Testes were dissected and placed in 100 μl of incubation media as quickly as possible following dissection. Incubated testes were left in open tubes in darkness at room temperature for 4 hours. Because 4 hours are longer than the standard S phase of the early male germ line (*72*, *84*, *110*–*112*), all S phase cells at the end of the incubation should have been exposed to the inhibitor for the entirety of their current S phase.

For EdU incorporation, 20 μM EdU was added to the incubation media for the last 15 min of the drug incubation before tissue fixation. Only cells in early– to mid–S phase were used for quantifications, as denoted by EdU staining covering all or most of the nucleus. Cells with focal EdU signal, indicative of late S phase, were excluded to avoid skewing of the data. Germ cells were determined by endogenously tagged Vasa-mApple signals. Following imaging, EdU incorporation was quantified by measuring the mean EdU signal intensity in EdU-positive germline nuclei and subtracting the backsground measured from the nearby EdU-negative cells. When a cyst was considered, only one nucleus from each cyst was measured as one data point. Data shown in fig. S3B are both separated by cell stage and combined to consider all cells together.

### Generation of chromatin fibers from the *Drosophila* male germ line

Chromatin fibers were prepared as previously described (*16*, *20*). Briefly, after adding EdU to the testis samples and incubating for 15 min, lysis buffer was added [100 mM NaCl, 25 mM tris-base, and 0.2% Joy detergent (pH 10)]. The testis tip was then microdissected on the slide, and the rest of the testis was removed. Cells were allowed to fully lyse for approximately 5 min, and then a sucrose/formalin (1 M sucrose; 10% formaldehyde) solution was added and left for 2 min to incubate, before a cover slip was gently placed on the top. The slide was then transferred to liquid nitrogen for 2 min before the cover slip was removed. The slide was then transferred to 95% ethanol for 10 min at −20°C in a freezer. Afterward, the slide was fixed in 1% PFA for 1 min. Samples were washed three times in a Coplin jar with 1× PBST followed by blocking the sample with 3% BSA in 1× PBST for 30 min. Primary antibodies were then added for overnight incubation in a humidity chamber at 4°C. To assess histone asymmetry, anti-PCNA, anti-H3K27me3, and anti-GFP primary antibodies were added to chromatin fibers from the testes from the males with the following genotypes: each of the drivers (*nos-Gal4* itself, *nos-Gal4*Δ*VP16; bam-Gal80* combination, or *bam-Gal4* itself) crossed with *UASp-FRT-H3-EGFP-FRT-H3-mcherry* without *hs-flp*. For *cdc45-mCherry; DNA Polymerase-HA* fibers, mCherry and HA primary antibodies were used. After the incubation with the primary antibodies, the slides are washed in a Coplin jar with 1× PBS. Then, the secondary antibodies were added and incubated for 2 hours at room temperature in a humidity chamber. The click chemistry was performed to label EdU following the manufacturer’s instruction. When DNA needs to be labeled, Hoechst is included at 1:1000 to stain the samples. In addition, for samples that need DNA labeling, ProLong Gold Antifade Mountant with DNA Stain 4′,6-diamidino-2-phenylindole (DAPI; Thermo Fisher Scientific, catalog no. P36931) was used. For samples that do not need DNA labeling, ProLong Diamond mounting media without DAPI (Thermo Fisher Scientific, catalog# P36961) was used.

For fibers labeled with Cdc45-mCherry, after EdU incubation, the testis tip was once again microdissected but then placed in a collagenase/dispase solution to generate a cell suspension. To obtain fibers from replicative somatic cells, larval imaginal disks were dissected and placed in collagenase/dispase. After obtaining cell suspensions, cells were centrifuged onto a glass slide at 91.5 g for 4 min using a Thermo Fisher Scientific Shandon Cytospin 3. Slides were then placed in a 50-ml conical tube filled with the chromatin lysis buffer described above. After 5 min in lysis buffer, it was slowly drained through a hole in the bottom of the conical vial to create a slow, continuous pulling force on the fibers, resulting in more stretched fibers. After the buffer drained, the normal protocol continued from the sucrose/formalin step described above.

### Sequential labeling using EdU and BrdU analogs on DNA fibers

After sample dissection, 20 μM EdU was added for a 10-min incorporation, followed by washing out EdU. BrdU was subsequently added for another 10 min. After this sequential labeling, DNA fibers were prepared using the same procedure as described above for chromatin fibers, with the exception of using a different lysis buffer to strip proteins from the DNA [200 mM tris-HCl (pH 7.5), 50 mM EDTA, and 0.5% SDS]. The fibers were then treated with 1 M HCl for 30 min at room temperature to expose the incorporated BrdU. After washing with 1× PBST, BrdU antibodies were added for incubation overnight at 4°C in a humidity chamber. Secondary antibodies against the BrdU primary antibodies were then added for 2 hours at room temperature in a humidity chamber. The click reaction to recognize EdU was performed subsequently along with Hoechst incubation at 1:1000. Samples were then mounted in ProLong Diamond mounting media with DAPI.

The EdU-positive DNA fibers representing regions that undergo DNA replication during EdU pulse (and thus have EdU on at least one side) were used for subsequent analyses as shown in Fig. 5B. Here, the BrdU signal was not used as a criterion for fiber selection; thus, some fibers are BrdU negative if there is no active DNA replication within the EdU pulsing time.

### Identifying and imaging replicative DNA fibers and chromatin fibers

All DNA fibers and chromatin fibers in this study were imaged with the Airyscan mode on a Zeiss LSM800 using a 63× oil immersion lens. Germline-derived chromatin fibers were identified using the H3-EGFP signal expressed with different germ cell–specific drivers or driver combination. Replicative regions were identified by both PCNA and EdU signals or the presence of Cdc45, DNA polymerase, and EdU. Fiber regions with detectable separation between sister chromatids were imaged and analyzed. Quality controls to select appropriate chromatin fiber regions for further analyses included fiber length, shape, and the molecular specificity of signals. For example, for quantifying old histone-enriched H3K27me3 with strandedness information, the EdU labeled fibers positive with PCNA, H3-EGFP, and H3K27me3 signals were used. For analyzing the Cdc45 signals with DNA polymerases, fibers with EdU-labeling regions, clear Cdc45, and anti-HA signals were used.

For sequential EdU- and BrdU-labeled DNA fibers, two patterns were imaged and quantified at DNA regions that replicate during the EdU pulse (thus incorporating EdU on at least one side of the duplicated sister chromatids): first, regions with clear sister chromatid separation with Hoechst and EdU signals but no discernable BrdU signal; second, regions with clear sister chromatid separation with clear Hoechst, EdU, and BrdU signals. For detailed description of the analyses of sister chromatids using chromatin fibers, refer to (*16*, *20*).

DNA and chromatin fiber methods have evident technical limitations that are important to consider. Because no consistent stretching factor is applied (with the exception of the DNA fibers carrying Cdc45-mCherry), we cannot calculate the nucleotide length present in these images. In addition, these fibers have no sequence-specific identifiers, so while we can infer the epigenetic environment of the chromatin fibers (from the presence of H3K27me3), these sample a subset of unknown loci. Last, while care is taken to only select clear replication bubbles (one single strand becomes two separate strands and lastly returns to one single strand), there is no way to eliminate the possibility of selecting two distinct fibers that have rested on top of each other at all but one location of separation, appearing as a replication bubble. However, we expect this exact overlap to be rare, and the repeatability of fiber data from this study and our previous publication (*16*, *20*) suggests that the vast majority of bubbles measured is an authentic sample of replication bubbles present in replicative cells at the time of cell lysis.

### Quantification of DNA fibers and chromatin fibers

All images were analyzed using FIJI software, and this was done blind, without knowing the experimental group of images while analyzing. To quantify the asymmetry between sister chromatids, line plots were drawn on both strands, using the PCNA-enriched side to denote the lagging strand. Most fibers have relatively short separable regions (≤2 μm), for which the entire fiber was used for quantification. For fibers with longer separable regions (>2 μm), they were divided into 2-μm-long nonoverlapping segments along the length of the chromatin fiber, and each of them was used for analyses. The region with no overlap with any of the chromatin fibers was used as background signal for subtraction from the measured signals from both strands. The ratio of signals = log_2_ (leading strand signal − background signal)/(lagging strand signal − background signal).

For DNA fiber analysis, all detectable replication bubbles labeled with EdU were first considered and categorized as symmetric (EdU only on both strands), weakly asymmetric (both EdU and BrdU but less than a twofold asymmetry on both strands), or strongly asymmetric (greater than twofold difference for at least one of the signals). Fibers with both labels (i.e., replicated during both the EdU pulse and five min later during the BrdU pulse) were considered for further analysis. Because of the lack of protein retention on DNA fibers, there is no strandedness indicator such as PCNA. As such, the strand with higher BrdU signals was used as the reference strand, allowing EdU signal to be independently measured, which could be on the same or the opposite strand. All quantifications were performed similar to the chromatin fibers. For example, after background subtraction, (BrdU on BrdU high side)/(BrdU on BrdU low side) to quantify the balance of BrdU and (EdU on BrdU high side)/(EdU on BrdU low side) to quantify the ratio of EdU on the strands relative to the BrdU side. The log_2_ of these ratios is graphed in Fig. 5C and fig. S5B.

For the fibers labeled with Cdc45 and EdU, active replication forks were identified by EdU tracts extending from Cdc45 foci. These were classified as either bidirectional (two Cdc45 foci flanking EdU, as in fig. S5G) or unidirectional if consecutive forks displayed movement in the same direction (EdU extending from a single Cdc45 focus as in fig. S5H). For the Cdc45- and DNA polymerase–labeled fibers, the distance between Cdc45 signal and the HA signal (labeling either Polα or Pole) was quantified from the center of the Cdc45 focus to the nearest HA signal.

### A quantitative assay for chromosomal condensation state

We used an area-based method to monitor the chromosomal condensation state as previously described (*22*), using a dual-color histone transgene *UASp-FRT-histone-EGFP-FRT-histone-mCherry*. A maximum intensity projection was generated for old H3– (EGFP) and new H3– (mCherry) enriched areas. The intensity of each pixel was determined and scaled individually, setting the minimum intensity to 0 and the maximum to 65,535 (a 16-bit range). We monitored the pixels across the image with a threshold of 35% of the maximum intensity. Condensation kinetic profiles were generated to compare old H3- versus new H3-enriched regions by calculating the percentage of pixels above the threshold (the condensation parameter). Relative compaction index was measured and plotted by taking a ratio of the percentage of pixels of the new H3-enriched to the old H3-enriched regions as described previously (*22*).

### Statistics and reproducibility

For all comparisons between two groups, Mann-Whitney tests were used unless otherwise noted. For one-group datasets, one-sample *t* test was used with a null hypothesis that the data are symmetrically distributed (e.g., ratio = 1 for datasets without logarithmic transformation, log_2_ = 0 for logarithmically transformed data).
