# Supplementary material for: Increased levels of lagging strand polymerase α in an adult stem cell lineage affect replication-coupled histone incorporation
Source: Sci Adv. 2025 Feb 28;11(9):eadu6799. doi: 10.1126/sciadv.adu6799 (PMC11870066; doi:10.1126/sciadv.adu6799)
Supplement: Supplementary file 1 — Figs. S1 to S5 Tables S1 to S28 [file sciadv.adu6799_sm.pdf]

Science Advances
AAAS

Supplementary Materials for

Increased levels of lagging strand polymerase α in an adult stem cell lineage affect replication-coupled histone incorporation

Brendon E. M. Davis *et al.*

Corresponding author: Xin Chen, xchen32@jhu.edu

Sci. Adv. **11**, eadu6799 (2025)
DOI: 10.1126/sciadv.adu6799

This PDF file includes:

Figs. S1 to S5
Tables S1 to S28

Fig. S1

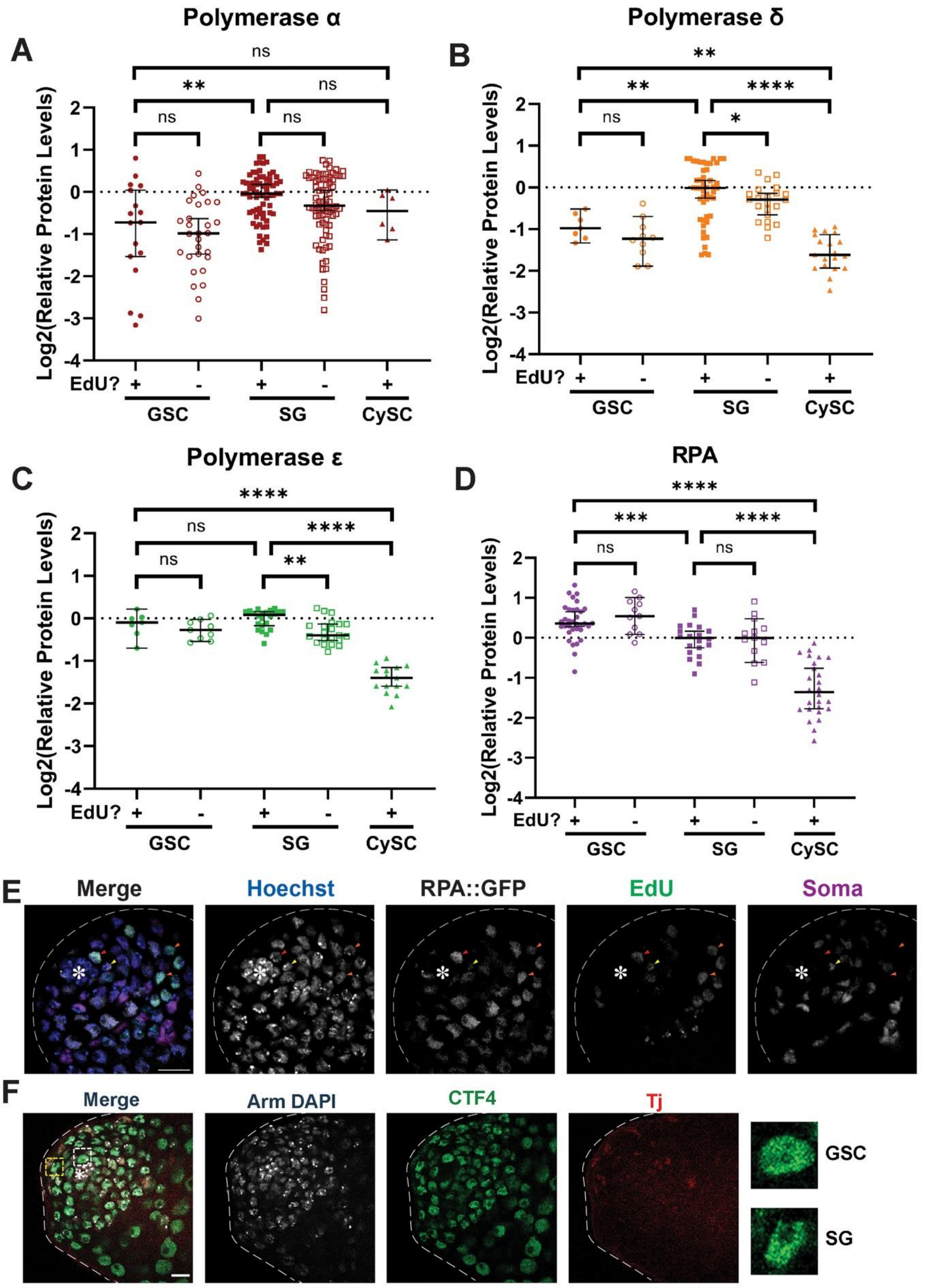

Polymerase α
A
ns
**
ns
ns
ns
Log2(Relative Protein Levels)
EdU? + - + - +
GSC
SG
CySC
Polymerase δ
B
**
**
****
ns
*
Polymerase ε
C
****
ns
****
ns
**
RPA
D
****
***
****
ns
ns
E
Merge
Hoechst
RPA::GFP
EdU
Soma
F
Merge
Arm DAPI
CTF4
Tj
GSC
SG

Figure S1: Expression patterns of replication machinery components with respect to S-phase. (**A-D**) Quantification of the relative protein expression of (**A**) Polα-HA, (**B**) Polδ-HA (**C**) Polε-HA, and (**D**) RPA-EGFP, separated by cell type and whether the cell is in S-phase as marked by EdU. GSCs, SGs, and EdU-positive somatic cyst stem cells (CySCs) shown. Polα-HA medians: EdU+ GSC= -0.72 (n=17), EdU- GSC= -0.98 (n=29), EdU+ SG= -0.04 (n=69), EdU- SG= -0.33 (n=77), and EdU+ CySC= -0.45 (n=6). See Table S2 for Polα-HA quantification. Polδ-HA medians: EdU+ GSC= -0.98 (n=7), EdU- GSC= -1.23 (n=10), EdU+ SG= -0.01 (n=48), EdU- SG= -0.29, and EdU+ CySC= -1.62 (n=19). See Table S3 for Polδ-HA quantification. Polε-HA medians: EdU+ GSC= -0.10 (n=7), EdU- GSC= -0.27 (n=9), EdU+ SG= 0.08 (n=22), EdU- SG= -0.40 (n=21), and EdU+ CySC= -1.40 (n=15). See Table S4 for Polε-HA quantification. RPA-EGFP medians: EdU+ GSC= 0.36 (n=34), EdU- GSC= 0.54 (n=11), EdU+ SG= 0.00 (n=20), EdU- SG= 0.00 (n=13), and EdU+ CySC= -1.36 (n=26). See Table S5 for RPA-EGFP quantification. Median± 95% confidence interval shown. Mann-Whitney test, ****: $P < 10^{-4}$, ***: $P < 10^{-3}$, **: $P < 0.01$, *: $P < 0.05$, ns: not significant. (**E**) Representative image of *Drosophila* testis expressing RPA-EGFP transgene under its own promoter. Yellow arrowhead: EdU-positive GSC, orange arrowhead: EdU-positive SG, red arrowhead: EdU-positive CySC. Hoechst (blue), RPA-EGFP (white), EdU (green), and the somatic marker Traffic Jam (Tj, purple). Scale bar: 10µm. (**F**) Image of endogenous CTF4-GFP using knock-in strategy (Materials and Methods): DAPI (white), Arm (white, a marker for hub cells), CTF4-GFP (green), and the somatic marker Tj (red). Scale bar: 10 µm.

Fig. S2

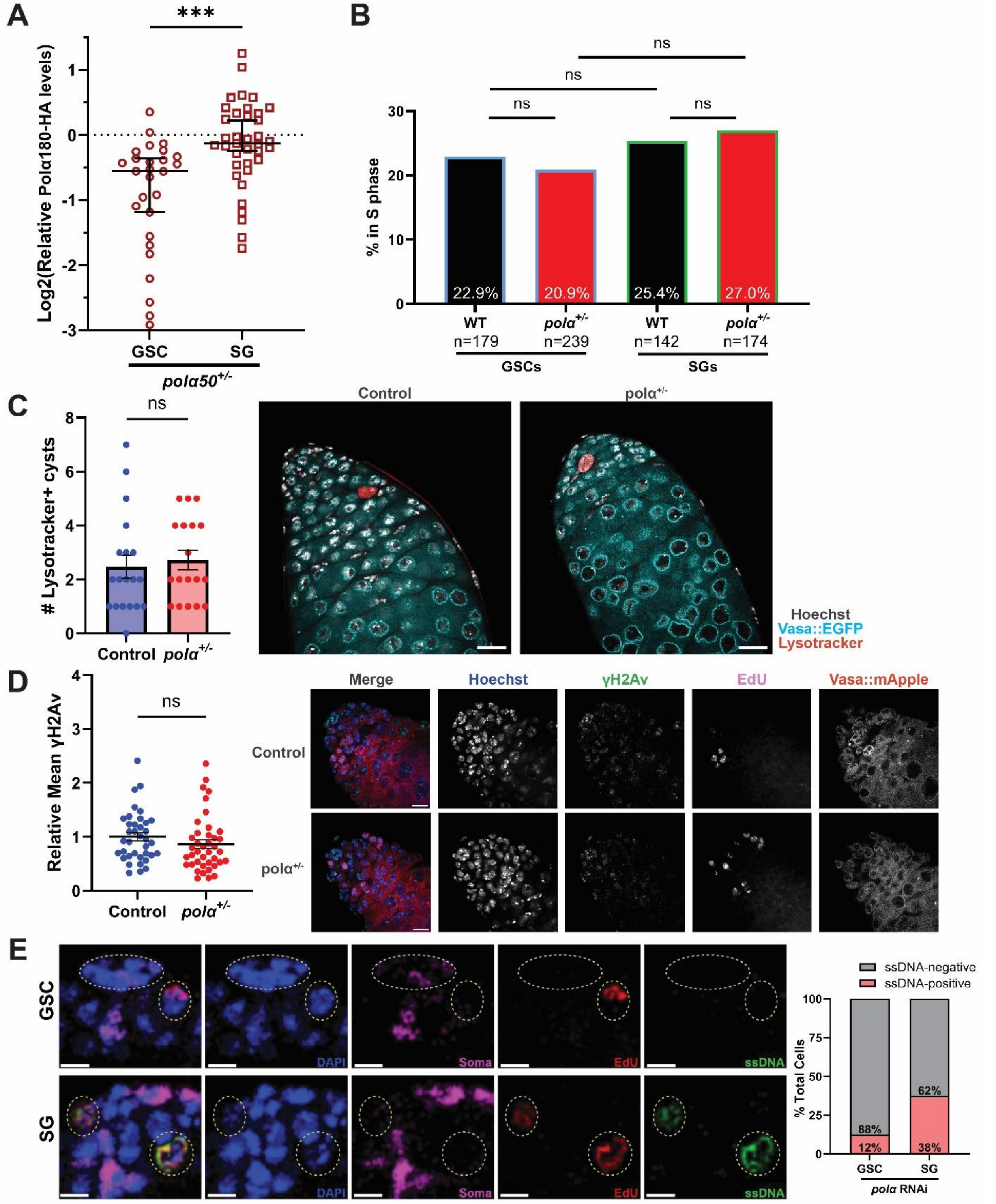

Figure S2: Effects of genetically depleting Polα. (**A**) Quantification of Polα180-HA protein levels in *polα50*$^{+/-}$ GSCs and SGs, normalized to the average level for SGs. Medians: *polα50*$^{+/-}$ GSCs= -56 (n=27), and *polα50*$^{+/-}$ GSCs = -0.13 (n=37). See Table S8 for details. Median± 95% confidence interval shown. Mann-Whitney test. (**B**) S-phase index of GSCs and SGs, calculated

by the percentage of EdU-positive cells/cysts divided by the total number of cells/cysts observed. See Table S9 for details. Chi-square test. (**C**) Quantification and representative images of control and *polα*$^{+/-}$ testes labeled with Lysotracker to indicate germline cell death. Each datapoint is one testis, quantified by the number of dying germline cysts observed through the whole early germline region. Control: 2.47± 0.43 (n=19), and *polα*$^{+/-}$: 2.72± 0.36 (n=18). See Table S10 for details. Mean± SEM shown. Student's t-test. For images: Hoechst (blue), endogenous germline marker Vasa-EGFP (green), and Lysotracker (red). Scale bars: 10μm**.** (**D**) Quantification and representative images of control and *polα*$^{+/-}$ testes immunostained for γH2Av. Mean γH2Av levels were quantified over one entire nucleus for each EdU-positive 4-cell SG identified. Control: 1.00± 0.08 (n=37), and *polα*$^{+/-}$: 0.87± 0.08 (n=40). See Table S11 for details. Mean± SEM shown. Student's t-test. For images: Hoechst (blue), anti-γH2Av (green), EdU (magenta), and endogenous Vasa-mApple (red). Scale bars: 10μm**.** (**E**) Germline-specific RNAi knockdown of *polα* yields significant germ cell death. Specific knockdown of *pola* in the germline of adult testes (see Materials and Methods) results in increased single-stranded DNA (ssDNA, green), a cell death marker (*61*), in S-phase germ cells. See Table S12 for details. For images: DAPI (blue), a somatic cell-enriched marker Traffic jam (Tj, magenta) (*109*), EdU (red). EdU-positive cells circled with dotted line (in both GSC and SG panels). Hub: large dotted circle (in GSC panel only). Scale bars: 5μm. ***: $P< 10^{-3}$, ns: not significant.

Fig. S3

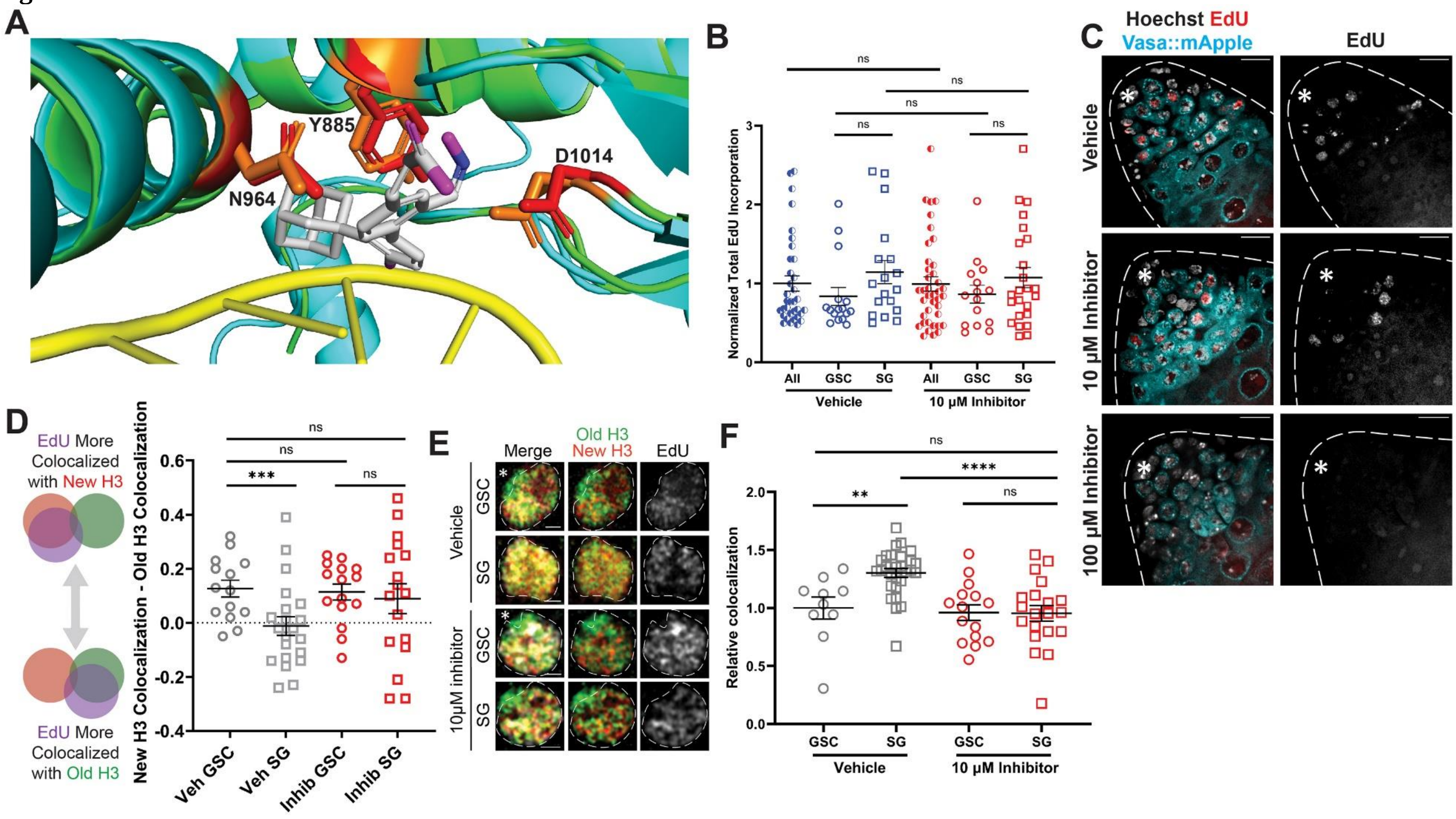

A
Y885
N964
D1014
B
ns
Normalized Total EdU Incorporation
All
GSC
SG
Vehicle
10 μM Inhibitor
C
Hoechst EdU
Vasa::mApple
EdU
Vehicle
10 μM Inhibitor
100 μM Inhibitor
D
EdU More Colocalized with New H3
EdU More Colocalized with Old H3
New H3 Colocalization - Old H3 Colocalization
***
Veh GSC
Veh SG
Inhib GSC
Inhib SG
E
Merge
Old H3
New H3
EdU
Vehicle
10μM inhibitor
F
**
****
Relative colocalization

Figure S3: Low concentration of Polα180 (or PolA1) inhibitor partially inhibits Polα while permitting DNA replication and affects histone colocalization in SGs. (**A**) PyMOL structural alignment of human DNA Polα (green) with DNA (yellow) (structure: PDB 5IUD) and the AlphaFold prediction of *Drosophila* Polα180 (cyan). The Polα180 inhibitor (carbon: white, oxygen: magenta, nitrogen: blue) is shown at the binding location predicted by (*66*). The Polα180 residues predicted to interact with the inhibitor are conserved between mammals (red) and *Drosophila* (orange). (**B**) Quantification of total EdU incorporation in early-stage germ cells, treated with vehicle or Polα180 inhibitor for four hours, normalized to the mean of vehicle-treated cells. Vehicle treated cells (all): 1.00± 0.10 (n=34), vehicle GSCs: 0.84± 0.11 (n=16), vehicle SGs: 1.14± 0.15 (n=18), 10μM PolA1 inhibitor treated cells (all): 0.99± 0.09 (n=39), inhibitor GSCs: 0.86± 0.11 (n=15), inhibitor SGs: 1.08± 0.13 (n=24). See Table S13 for details. Mean± SEM shown. Student's t-test, ns: not significant. (**C**) Representative images of testes treated with vehicle or Polα180 inhibitor. In merged images: Hoechst (white), endogenous Vasa-mApple (cyan), EdU (red). Asterisk: hub. Scale bars: 10 μm. (**D**) Colocalization of EdU with new H3 versus old H3, expressed as the difference between the two colocalization values. Quantified for GSCs and SGs following 4-hour incubation with vehicle (Veh) or 10μM PolA1 inhibitor (Inhib). Vehicle GSCs: 0.13± 0.03 (n=14), vehicle SGs: -0.01± 0.03 (n=21), inhibitor GSCs: 0.11± 0.03 (n=15), inhibitor SGs: 0.09± 0.06 (n=17). See Table S14 for details. Mean± SEM shown. Welch's t-test. (**E**) Airyscan images of representative GSCs and SGs treated with vehicle or Polα180 inhibitor for four hours prior to clearance buffer treatment and fixation. Dotted line outlines the EdU-positive region. Scale bars: 1 μm. (**F**) Quantification of the correlation between old H3 and new H3 signals in only the EdU-positive region in S-phase nuclei. Vehicle GSCs: 1.00± 0.09 (n=10), vehicle SGs: 1.3± 0.04 (n=28), inhibitor GSCs: 0.96± 0.07 (n=15), inhibitor SGs: 0.95± 0.07 (n=20). See Table S15 for details. Mean± SEM shown. Mann-Whitney test. ***: $p<10^{-3}$, **: p<0.01, ns: not significant.

Fig. S4

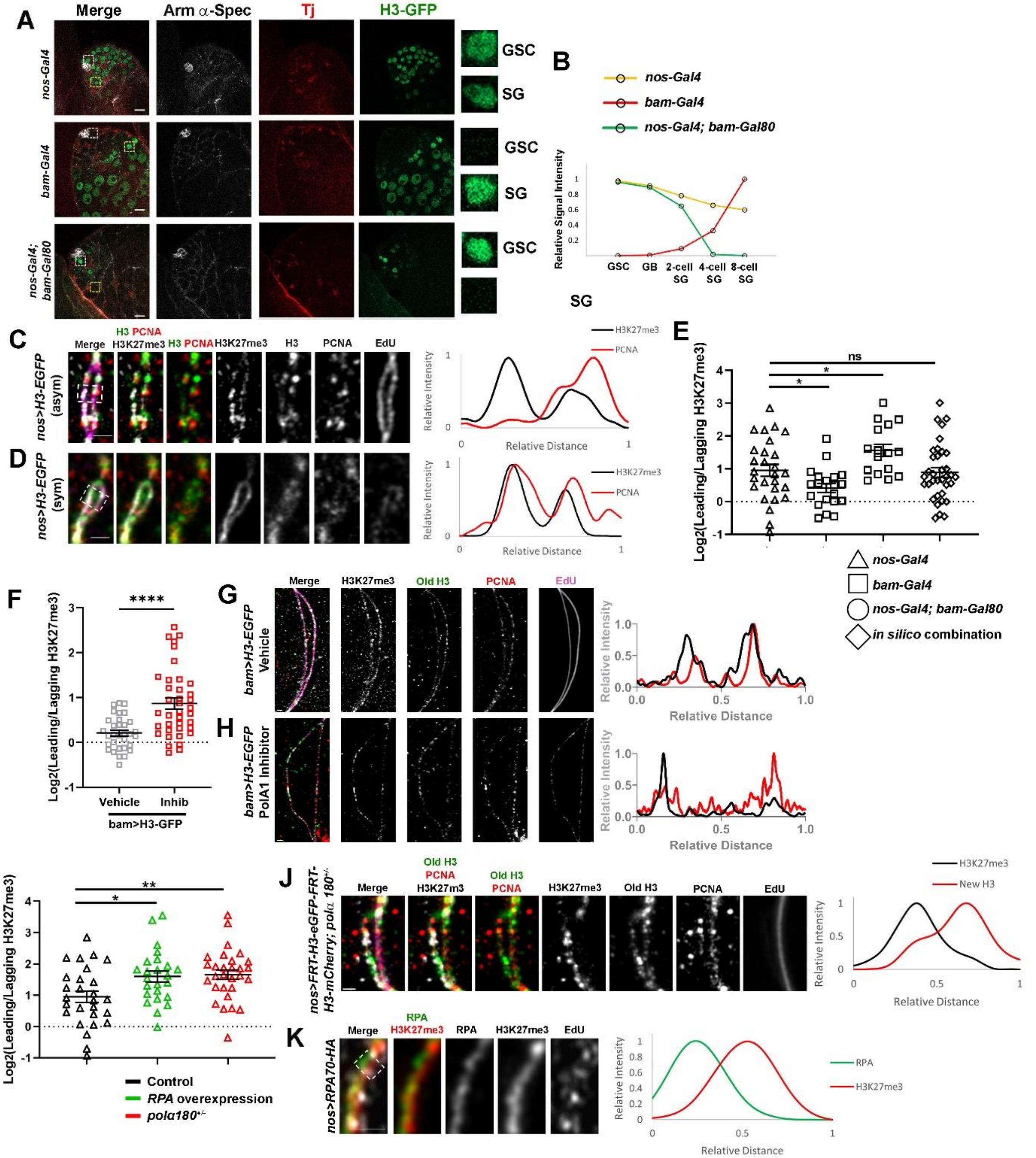

Figure S4: Reducing Polα or enhancing RPA levels increase old histone-enriched H3K27me3 asymmetries at the replication fork. (**A**) Images of *nanos-Gal4> UAS-H3-EGFP*, *bam-Gal4> UAS-H3-EGFP*, and *nos-Gal4ΔVP16; bam-Gal80> UAS-H3-EGFP*, Arm (white), H3-EGFP (green), and the somatic marker Tj (red). Asterisk: hub. Scale bars: 10 μm. (**B**) Quantification of the relative expression levels of H3-EGFP using each corresponding driver (n=3 for each genotyped testes). See Table S18 for details. Mean values shown. (**C**) An Airyscan image of representative asymmetric *nanos-Gal4>H3-EGFP* chromatin fiber. (**D**) An Airyscan image of representative symmetric *nanos-Gal4>H3-EGFP* chromatin fiber. In merged images (**C-D**): H3K27me3 (white), H3-EGFP (green), PCNA (red), and EdU (magenta). Images in (**C-**

D) are also accompanied by line plots showing the spatial distribution of H3K27me3 and PCNA signals from the indicated regions, respectively (white dotted outlined box). (**E**) Quantification of H3K27me3 asymmetry using chromatin fibers with *nanos-Gal4>H3-EGFP*, *bam-Gal4>H3-EGFP*, *nos-Gal4ΔVP16*; *bam-Gal80>H3-EGFP*, and an *in silico* combination of *bam-Gal4>H3-EGFP* and *nos-Gal4ΔVP16*; *bam-Gal80>H3-EGFP* in $\log_2$ scale: *nanos-Gal4>H3-EGFP*= 0.95± 0.18 (n=27), *bam-Gal4>H3-EGFP*= 0.42± 0.14 (n=20), *nos-Gal4ΔVP16*; *bam-Gal80>H3-EGFP*= 1.56± 0.19 (n=16), *in silico* combination of *bam-Gal4>H3-EGFP* and *nos-Gal4ΔVP16*; *bam-Gal80>H3-EGFP*= 0.93± 0.15 (n=36). See Table S19 for details. (**F**) Quantification of H3K27me3 asymmetry using chromatin fibers with *bam-Gal4>H3-EGFP* to label SG-derived chromatin, following 4-hour incubation of tissues with vehicle or 10μM PolA1 inhibitor in $\log_2$ scale: vehicle treated: 0.21± 0.07 (n=32), and inhibitor treated: 0.86± 0.12 (n=38). See Table S20 for details. (**G-H**): Representative images of *bam-Gal4>H3-EGFP* after incubation with (**G**) vehicle or (**H**) inhibitor. Scale bars: 1 μm. (**I**) Quantification of additional replication protein manipulations using a *nanos-Gal4* driven overexpression of *UAS-rpa70-HA* transgene and a P-element insertion allele of another Polα subunit gene (*polα180*) at a heterozygous background (*polα180*$^{+/-}$) in $\log_2$ scale: *nanos-Gal4>H3-EGFP*= 0.95± 0.18 (n=27), *nanos-Gal4>rpa70-HA*= 1.60± 0.17 (n=24), *nanos-Gal4>H3-EGFP; polα180*$^{+/-}$= 1.66± 0.15 (n=29). See Table S21 for details. (**J**) Airyscan image of *hs-flp*; *nanos-Gal4>FRT-H3-EGFP-FRT-H3-mCherry*; *polα180*$^{+/-}$: H3K27me3 (white), old H3 (green), new H3 (red), and EdU (magenta) in the merged image. (**K**) Airyscan image of *nanos-Gal4>rpa70-HA* chromatin fiber: EdU (white), RPA (green), and H3K27me3 (red) in the merged image. Images in (**J-K**) are also accompanied by line plots showing the spatial distribution of H3K27me3 and new H3 signals in (**J**), as well as H3K27me3 and RPA signals in (**K**) from the indicated regions, respectively (white dotted outlined box). Scale bar: 1 μm. All ratios: Mean± SEM. All statistics: Mann-Whitney test, ****: $P<10^{-4}$, **: $P< 0.01$, *: $P< 0.05$, ns: not significant.

Fig. S5

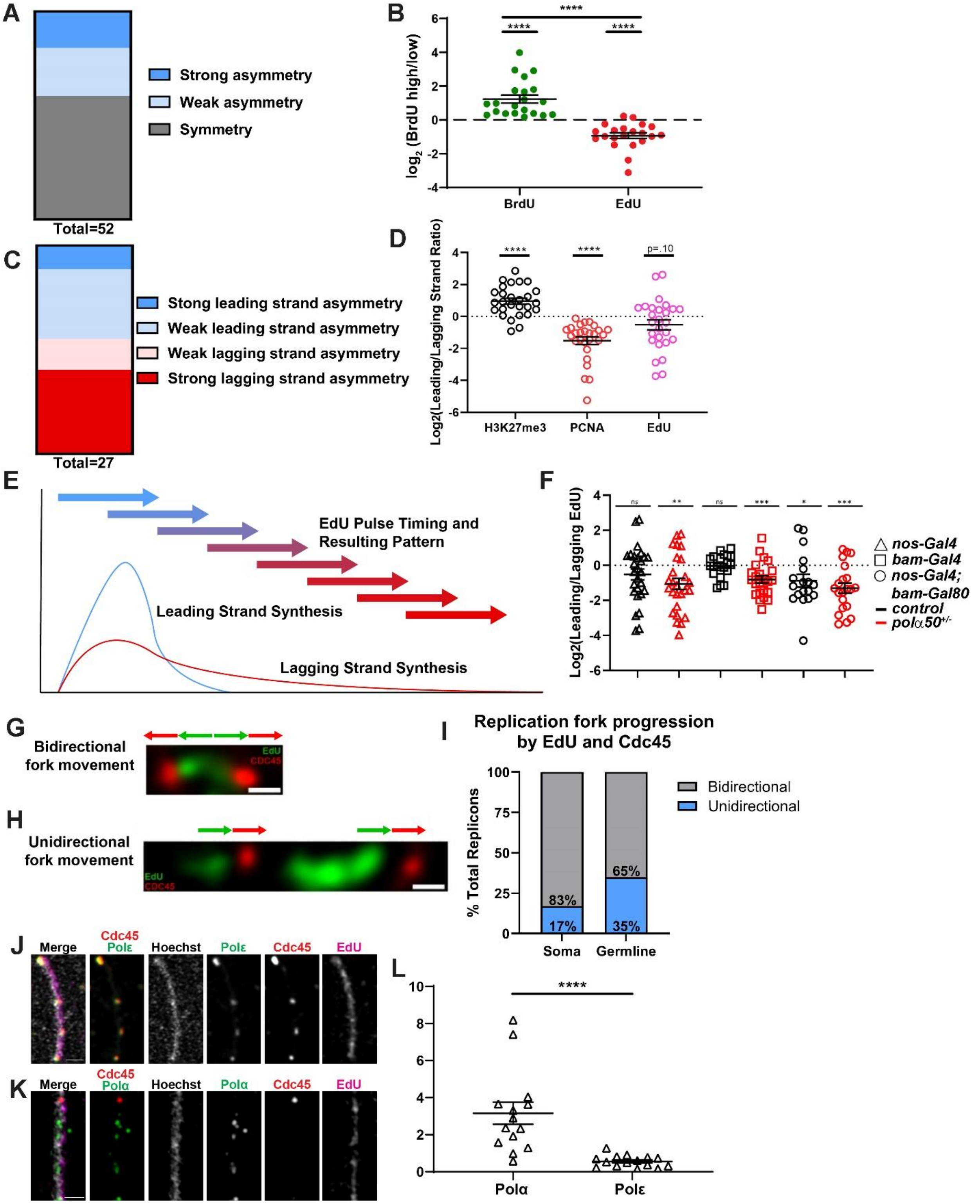

Figure S5: Visualization and quantification of delayed lagging strand synthesis. (**A**) Assessment of observed patterns on DNA fibers, wherein symmetric fibers refer to fibers with EdU on both strands, weak asymmetric fibers have both EdU and BrdU but less than a 2-fold asymmetry on both strands, while strong asymmetric fibers have a greater than 2-fold difference

for at least one of the signals (i.e., either EdU or BrdU). The distribution of the three categories are as follows: 60% symmetric, 23% weak asymmetric, 17% strong asymmetric. See Table S24 for details. (**B**) Log_2-scale 1D quantification of EdU and BrdU from the DNA fibers with both signals, where the positive side is the strand with higher BrdU and the negative side is the strand with higher EdU: log_2BrdU= 1.23± 0.23 (n=21), log_2EdU= -0.93± 0.17 (n= 21). See Table S22 for details. Mann-Whitney test for the comparison between two groups, one tailed t-test with a null hypothesis of log_2= 0 (symmetric pattern). (**C**) Assessment of EdU asymmetries wherein ≥ 2-fold are considered strong asymmetry, < 2-fold are considered weak asymmetry. The distribution of the three categories are as follows: 11% strong asymmetry toward the leading strand, 33% weak asymmetry toward the leading strand, 15% weak asymmetry toward the lagging strand, 41% strong asymmetry toward the lagging strand. See Table S25 for details. (**D**) Quantification of H3K27me3, PCNA, and EdU asymmetry from *nos-Gal4>H3-EGFP* labeled chromatin fibers using log_2 scale: log_2H3K27me3= 0.95± 0.18 (n=27), log_2PCNA= -1.52± 0.24 (n=27), log_2EdU= -0.52± 0.31 (n=27). See Table S26 for details. One tailed t-test with a null hypothesis of log_2= 0 (symmetric pattern). (**E**) A model of replication patterns that could explain the observed EdU patterns. (**F**) Quantification of EdU distribution on chromatin fibers labeled with H3-EGFP driven by the following drivers with regard to the strandedness using log_2 scale: *nanos-Gal4*= -0.52± 0.31 (n=27, *P*= 0.103, ns), *nanos-Gal4*; *polα50*$^{+/-}$= -1.06± 0.32 (n=26, *P*< 0.01), *bam-Gal4*= -0.03± 0.14 (n=21, *P*= 0.817, ns), *bam-Gal4*; *polα50*$^{+/-}$= -0.81± 0.21 (n=23, *P*< 10^{-3}), *nos-Gal4ΔVP16*; *bam-Gal80*= -0.88± 0.37 (n=18, *P*< 0.05), *nos-Gal4ΔVP16*; *bam-Gal80*; *polα50*$^{+/-}$= -1.31± 0.29 (n=21, *P*< 10^{-3}). See Table S23 for details. All ratios: Mean± SEM, one tailed t-test with a null hypothesis of log_2= 0 (symmetric pattern). (**G-H**) Representative images of (**G**) bidirectional fork progression, where EdU is flanked by Cdc45 on both sides, and (**H**) unidirectional fork progression, where consecutive forks display EdU with Cdc45 on only one side. Scale bars: 1μm. (**I**) Quantification of fork progression patterns in germline-derived versus soma-isolated chromatin fibers. Germline-derived fibers show a higher incidence of unidirectional fork progression (35%, n = 26) than soma-isolated fibers (17%, n = 12). See Table S27 for details. (**J-K**) Visualization of potentially asynchronous DNA strand syntheses: Airyscan image of representative chromatin fibers labeled with endogenous Cdc45-mcherry (red), EdU (magenta), Hoechst (white), along with (**E**) Polε-HA (green) or (**F**) Polα-HA (green). Scale bars: 1 μm. (**L**) Quantification of the distance along chromatin fibers from the center of the Cdc45 signal to the nearest signal of the DNA polymerase: Polα= 3.16± 0.60 μm (n= 14), Polε= 0.56± 0.08 μm (n= 15). See Table S28 for details. All ratios: Mean± SEM. Mann-Whitney test. ****: *P*< 10^{-4}, ***: *P*< 10^{-3}, **: *P*< 0.01, *: *P*< 0.05, ns: not significant.

Supplemental Tables

Table S1: Raw Data Related to Figure 1B (log_2 scale):

GSC Polα	SG Polα	GSC Polδ	SG Polδ	GSC Polε	SG Polε
-1.31257	0.478214	-0.53529	0.025996	-0.05733	0
0.309191	0.179521	-0.22863	0.522421	0.108252	-0.28951
-0.37796	0.34155	-0.82902	-0.28951	0.192645	-0.11704
-0.58645	-0.30091	-1.13936	-1.02647	0.036995	0.303069
0.282692	-0.70322	-1.25966	0.368388	-0.1375	0
-0.8576	-1.69149	-1.08278	0.172836	-0.33605	0.37707
-1.74014	0.843809	-1.02833	0.115881	-0.4339	0.272372
-1.29675	-0.41973	-1.78322	-0.35509	0.125531	-0.2007
-1.39827	-1.2292	-1.78322	-0.73696	-0.01886	-0.3599
-1.09303	0.909612	-1.46129	-0.1375	-0.22239	-0.48543
-2.0808	0.249741	-0.35695	0	-0.56635	-0.48543
-2.41359	-1.2475	-0.61329	0.466568	0.159479	-0.38414
-2.87359	-0.29352	-1.19826	0	0.347923	-0.28951
-1.72364	-0.34207	-0.97586	-0.1375	0.036995	-0.03797
-0.10579	-0.23503	-0.82902	-0.2115	-0.24442	-0.4339
-0.16098	-0.27503	-1.08278	-0.49596	-0.4088	-0.5119
-1.39736	0.492338	-0.46129	-0.06711	-0.46667	-0.65208
-0.9895	0.601421		-0.67283	-0.42182	-0.31259
-0.65638	0.397087		-0.08927	-0.5771	0.036995
-0.59786	-0.77119		-0.65703	-0.73275	-0.26679
-1.33687	0.605119		-0.78587		-0.05733
-0.58599	0.530551		-0.2863		0.192645
-0.87321	0.278698		-0.08092		0.287803
-1.41299	-1.55611		0		0.318177
-0.55373	0.58095		0.172836		0.256775
-0.8475	-0.91912		0.125532		0.303069
-1.17455	0.525471		0.076621		0
-0.84107	0.469582		0.327165		-0.17932
-0.06675	0.312027		0.149378		0.272372
0.17612	0.67072		-0.10893		-0.03797
0.604322	1.002294		0.327165		-0.24442
-0.64542	0.194597		0.558491		-0.1375
-0.10228	-0.09784		0.172836		-0.45943
-0.49791	0.424877		-0.1375		-0.1375
-2.1112	0.425666		0.742202		0.36257
-1.29082	0.549028		0.862497		0.241008
-2.74306	-0.42891		0.149378		0.125531
	-0.68767		0.149378		0.192645

	-1.09098		0.241008		0.287803
	0.16498		-0.05344		0.347923
	-0.26264		-0.1964		0.256775
	-0.3827		0.7578		0.018616
	-0.2624		0.77323		0.287803
	0.338635		0.803603		
	0.196455		0.742202		
	0.017312		0.803603		
	-0.28549		0.59368		
	-1.18792		0.833351		
	0.486708		0.77323		
	0.583987		0.263035		
	-0.38879		-1.45943		
	-0.38339		-1.38904		
	0.96703		-1.02647		
	-1.51239		-1.32193		
	-0.35785		-0.65208		
	0.16457		-0.61143		
	-0.42837		-1.08092		
	-0.91937		-0.92338		
	-0.16938		-0.53343		
	-0.24721		-1.45223		
	-0.32259		0.412413		
	-2.00388		0.847997		
	-0.63923		0.347924		
	-0.93225		0.051531		
	-2.37993		-0.53343		
	-0.04774		-0.10893		
	-1.16026		-0.6939		
	-0.20723		-0.78136		
	-1.70132		0.788496		
	-0.61486		0.803603		
	-0.65722				
	0.211628				
	0.056212				
	-2.66654				
	-0.89585				
	-1.06545				
	-0.15041				
	-0.32111				
	0.264392				

	-0.59622				
	0.09556				
	0.560539				
	0.63656				
	0.314024				
	-0.00704				
	-2.17927				
	-1.23407				
	-0.51216				
	-0.34315				
	-0.6354				
	-0.18619				
	0.56031				
	0.374148				
	0.559079				
	0.300244				
	-0.49019				
	-1.13866				
	0.79303				
	0.440277				
	0.96703				
	0.741985				
	-1.01161				
	-0.22325				
	-0.27899				
	0.875468				
	0.510143				
	0.5178				
	0.713183				
	0.633194				
	0.886434				
	0.439395				
	0.835981				
	0.491163				
	0.432999				
	0.097461				
	0.06878				
	0.095233				
	0.007007				

Raw Data Related to Figure 1B (continued):

GSC Cdc45	SG Cdc45	GSC Ctf4	SG Ctf4	GSC RPA	SG RPA
-0.40234	-0.18844	0.623142	0.951972	-0.87366	-0.20198
0.118644	-0.34792	0.609015	-0.50849	0.177189	-0.67186
-0.18844	-0.10692	0.171094	-0.1383	-0.43521	-0.92266
0.166649	0.014646	-0.35686	-0.74374	0.263834	0.532229
0.111576	-0.02975	0.066299	0.490909	-0.20358	0.001061
-0.25716	-0.18844	-0.04728	-0.26161	0.325477	0.268228
0.014646	0	0.243402	0.802319	-0.10151	-0.0562
0.032029	-0.00295	0.353389	-0.05505	0.891319	0.241818
0.166649	-0.32931	0.204584	0.254205	1.076262	0.136621
-0.50619	0.179706	0.573534	-0.79276	0.682312	0.141762
0.192645	0.099536	-0.00298	0.196288	0.335832	0.670859
0.057715	0.20547	0.053226	0.364334	0.257006	0.292367
0.192645	0.179706	0.127204	-0.37851	0.398501	-0.27638
-0.31093	-0.0148	0.210356	0.278883	0.493074	-0.38769
-0.20532	0.304153	0.003056	0.452054	0.269012	0.011147
0.099536	-0.32931	-0.30331	-0.29025	0.43558	-0.22151
-0.50773	0.166649	-0.08452	-0.07451	0.732926	0.034061
0.166649	0.140178	0.275981	0.105654	0.18717	-0.56154
0.071791	0.126757	0.103751	0.704144	0.626317	-0.0976
0.113211	0.065614	-0.18246	-0.3556	0.66488	-0.5009
-0.32931	-0.44479	0.090752	-0.16395	0.305203	-1.13669
		-0.35899	0.116547	0.266347	0.879537
		-0.01312	0.362708	1.291699	-0.66228
		0.064503	0.040772	1.097938	0.207906
		-0.1226	-0.06534	0.98986	0.448822
		-0.39215	-0.35847	0.198014	-0.13434
		0.262724	0.742378	0.377783	0.132609
		-0.01062	-0.31665	0.696822	0.088147
		0.123721	0.370067	0.313681	0.575382
		-0.19653	-0.4246	0.681646	-0.07006
		0.134335	-0.49635	-0.09088	-0.35345
		-0.07488	0.011934	0.109763	-0.6426
		0.118006	-0.29622	0.375408	-0.02943
		0.68878	0.201033	-0.18122	
		-0.36554	0.608532	0.517934	
		-0.24475	-0.42571	0.485186	
		0.013141	0.241597	0.67638	
		-0.44443	0.268592	0.979879	
		-0.32045	-1.04457	0.882713	

		-0.27927	-0.01203	0.058793	
				-0.1485	
				1.132868	
				0.813884	
				0.228886	
				0.059759	

Table S2: Raw Data Related to Figure S1A (log_2 scale):

Polα				
GSC		SG		CySC
EdU+	EdU-	EdU+	EdU-	EdU+
-1.44717	-1.90454	0.356556	0.472462	0.048919
0.174584	-0.72105	0.298392	-1.13991	-0.14875
-0.51256	-0.99221	-0.03715	-0.57353	-0.874
0.148085	-1.87475	-0.06583	0.343607	-0.76
-3.1605	-1.43136	-0.03937	0.044913	-1.13775
-1.22764	-1.53287	0.214758	0.206943	-0.07574
-1.85824	-2.21541	0.681107	-0.43552	
-1.53197	-2.5482	0.214758	-1.8261	
-0.73246	-3.0082	0.681107	-0.55434	
-0.72059	-0.2404	-0.83782	-1.36381	
0.041513	-0.29559	0.709203	0.115134	
-2.87766	-1.12411	0.357731	-1.38211	
-2.94317	-0.79099	0.466813	-0.42813	
0.803276	-1.47147	0.470512	-0.47668	
0.378616	-1.5476	0.144091	-0.36963	
-0.48798	-0.68834	-1.05373	-0.40964	
-0.32894	-0.98211	0.17742	0.26248	
	-1.30915	0.832423	-0.9058	
	-0.97568	0.05999	0.395944	
	-0.20136	-0.82228	-1.69071	
	-0.78003	-1.22559	0.446343	
	-0.23689	-0.39725	0.390864	
	-0.63252	-0.51731	0.334975	
	-2.24581	0.204028	0.536113	
	-1.42543	-0.11729	-0.23245	
	0.43581	-0.4201	0.29027	
	-0.08129	-0.518	0.29106	
	0.115093	0.832423	0.414421	
	-0.01703	-0.49245	-0.56351	
		-1.06686	0.030373	
		-0.74946	-0.39701	
		0.07702	-1.32252	
		-0.0784	0.352101	
		-1.20005	0.44938	
		-0.28501	-0.52339	
		-0.45572	-1.64699	
		0.501953	-0.56298	

		0.179417	-1.05398	
		-1.36868	-0.30398	
		-0.3208	-0.38182	
		0.425702	-0.45719	
		0.239542	-2.13848	
		-0.6248	-0.77384	
		-1.14622	-2.51454	
		0.701374	-0.18235	
		0.356556	-1.29487	
		0.298392	-0.34184	
		-0.03715	-1.83593	
		-0.06583	-0.79182	
		-0.03937	-2.80115	
		-0.14163	-1.03045	
		0.199401	0.129785	
		0.406905	-0.73083	
		-0.50172	-0.03905	
		0.178703	0.425932	
		-0.0855	-0.14165	
		0.245838	-2.31388	
		0.152038	-0.64676	
		0.195614	-0.77001	
		0.308592	0.424472	
		0.46752	0.165637	
		0.094398	-1.27326	
		-0.77703	0.658423	
		-0.00062	0.305669	
		-0.48534	-0.35786	
		-0.58667	-0.4136	
		-0.82902	0.740861	
		-0.68829	0.375537	
		-0.12879	0.383193	
			0.578576	
			0.498587	
			0.751826	
			0.304788	
			0.047	
			-0.23128	
			-0.22664	
			-0.32821	

Table S3: Raw Data Related to Figure S1B (log_2 scale):

Polδ				
GSC		SG		CySC
EdU+	EdU-	EdU+	EdU-	EdU+
-0.515	-0.69878	-0.23906	-0.14055	-1.64804
-0.78029	-0.38523	-0.16022	0.355528	-1.07527
-1.33278	-0.95093	0.004544	-0.44592	-1.01058
-1.10887	-1.26308	-0.01972	-1.21117	-1.30991
-0.97784	-1.36498	-0.07704	0.198652	-1.04328
-1.21685	-1.20628	0.169625	-0.00086	-1.13022
-0.62584	-1.16704	-0.01543	-0.05257	-1.69135
	-1.89144	-0.25814	-0.52651	-1.56016
	-1.88577	0.160832	-0.89624	-2.19046
	-1.55565	0.405282	-0.29303	-1.9318
		0.01396	-0.15712	-2.46931
		-0.28188	0.297062	-1.61661
		0.577201	-0.1602	-1.35318
		0.693995	-0.29515	-0.94428
		-0.00558	-0.37786	-1.93619
		-0.0117	-0.65954	-1.86051
		0.08959	-0.23665	-1.71506
		-0.21545	-0.83979	-1.29939
		-0.35042	-0.25849	-1.94649
		0.594493	-0.82546	
		0.607375	-0.95612	
		0.637241	-0.45349	
		0.577392		
		0.69281		
		0.420216		
		0.693995		
		0.600317		
		0.100005		
		-1.61221		
		-1.58406		
		-1.21146		
		-1.43206		
		-0.7857		
		-0.7658		
		-1.1954		
		-1.08279		
		-0.68509		

		-1.61934		
		0.244052		
		0.693995		
		0.177779		
		-0.10512		
		-0.70012		
		-0.27473		
		-0.83461		
		-0.92		
		0.629769		
		0.693995		

Table S4: Raw Data Related to Figure S1C (log_2 scale):

Polε				
GSC		SG		CySC
EdU+	EdU-	EdU+	EdU-	EdU+
-0.00701	-0.18987	0.060105	-0.13254	-1.1514
-0.1514	-0.02429	0.155262	-0.42205	-1.11392
-0.35493	0.060105	0.185636	-0.24958	-1.2294
-0.69889	-0.09555	0.124235	0.170529	-1.04177
0.026938	-0.27004	0.170529	-0.13254	-2.0774
0.215383	-0.46859	-0.13254	0.24453	-1.81436
-0.09555	-0.56644	-0.31186	0.139832	-1.39933
	-0.37696	0.139832	-0.33324	-1.2294
	-0.54135	-0.17051	-0.49244	-1.59197
		-0.37696	-0.61797	-1.59197
		-0.27004	-0.61797	-1.59197
		-0.59197	-0.51668	-1.75547
		0.23003	-0.42205	-0.93989
		0.108468	-0.17051	-1.59197
		-0.00701	-0.56644	-1.35493
		0.060105	-0.64444	
		0.155262	-0.78462	
		0.215383	-0.44513	
		0.124235	-0.09555	
		-0.11392	-0.39933	
		-0.27004	-0.18987	
		0.155262		

Table S5: Raw Data Related to Figure S1D (log_2 scale):

RPA				
GSC		SG		CySC
EdU+	EdU-	EdU+	EdU-	EdU+
-0.84727	0.544323	-0.1756	-1.1103	-1.41829
0.203579	0.511575	-0.64548	0.905926	-2.31473
-0.40882	0.70277	-0.89627	-0.63589	-2.57115
0.290223	1.006268	0.558618	0.234295	-0.49207
-0.17719	0.909103	0.027451	0.475211	-1.11266
0.351866	0.085182	0.294618	-0.10795	-0.76429
-0.07512	-0.12211	-0.02981	0.158999	-1.77007
0.917709	1.159257	0.268207	0.114536	-1.59748
1.102651	0.840273	0.16301	0.601771	-1.78302
0.708702	0.255275	0.168151	-0.04367	-1.00767
0.362221	0.086148	0.697248	-0.32706	-1.61975
0.283396		0.318756	-0.61621	-0.13162
0.42489		-0.24999	-0.00304	-0.30941
0.519463		-0.3613		-2.05788
0.295401		0.037536		-0.3641
0.461969		-0.19512		-1.30015
0.759315		0.06045		-0.48067
0.213559		-0.53515		-0.4436
0.652706		-0.07121		-1.79409
0.691269		-0.47452		-0.63619
0.331593				-1.72722
0.292736				-1.5909
1.318088				-1.85096
1.124327				-2.09709
1.016249				-1.26569
0.224403				-0.92506
0.404172				
0.723212				
0.34007				
0.708035				
-0.06449				
0.136152				
0.401798				
-0.15483				

Table S6: Raw Data Related to Figure 2C:

WT GSC	WT SG	*polα50*$^{+/-}$ GSC	*polα50*$^{+/-}$ SG
1.05	1.138636	0.986842	1.131579
1.009091	1.077273	1.013158	1.026316
0.940909	1.138636	0.957613	1.026817
0.942308	1.20858	1.052768	0.966263
1.127219	1.326923	0.927336	1.000865
0.927515	1.275148	1.031142	1.031142
1.08284	1.289941	1.031142	1.07872
0.920118	1.087209	1.012857	1.031142
0.994186	1.139535	1.087143	0.966263
1.005814	1.048246	0.944286	1.035467
1	1.039474	0.955714	1.161429
0.881579	1.372807		1.001429
0.995614	1.372807		1.018572
0.995614	1.232456		0.932857
1.004386	1.355263		0.852857
1.083333	1.47807		0.921429
1.039474			

Table S7: Raw Data Related to Figure 2F:

GSC			
Vehicle	2.5µM inhibitor	5.0µM inhibitor	10µM inhibitor
1.109244	1.092308	1.051546	1.086792
0.890756	1.015385	1.128866	1.041509
0.981132	1.107692	0.912371	0.739623
1.14717	1.092308	1.128866	1.056604
0.996226	0.938462	1.020619	0.981132
0.875472	1.046154	1.121212	1.116981
0.943089	0.984615	0.878788	1.071698
1.056911	1.076923	0.863636	1.14433
1.082474	1.046154	0.848485	1.06701
0.881443	1.097938	1.121212	1.097938
1.036082	1.113402	1.166667	1.175258
1.045455	1.097938	1.030303	1.075758
1.075758	1.06701		0.833333
0.80303	0.896907		1.015152
1.075758			1.136364

SG			
Vehicle	2.5µM inhibitor	5.0µM inhibitor	10µM inhibitor
1.092437	0.969231	0.943299	1.207547
1.243697	1.184615	0.974227	0.860377
1.327731	1	1.14433	1.116981
1.142857	1.230769	1.113402	1.056604
1.207547	1.107692	1.14433	1.011321
1.056604	0.876923	1.221649	1.177358
1.177358	0.969231	1.206186	1.086792
1.298113	1.138462	0.742424	1.132075
1.25283	1.2	1.19697	0.875472
1.011321	1.138462	1.272727	0.935849
1.298113	1.107692	1.19697	1.221649
1.071698	1.237113	0.772727	0.896907
1.298113	1.221649	0.863636	0.909091
1.086792	1.190722	0.939394	0.833333
1.14717	1.376289	1.287879	1.106061
1.170732	1.190722	0.863636	0.772727
1.235772	0.958763	1.227273	1.166667
1.284553	1.314433	1.060606	

1.283505	1.036082	1.212121	
1.268041	1.268041		
1.051546	0.927835		
1.237113	1.252577		
1.237113	1.175258		
1.082474			
1.391753			
1.333333			
1.333333			
1.212121			

Table S8: Raw Data Related to Figure S2A:

GSC	SG
-0.38546	0.41904
-0.52708	0.240114
-0.23704	-1.73748
-1.18428	0.287763
-0.42868	0.573615
-0.4469	1.040255
-0.15988	1.253657
0.038481	0.57805
-1.69258	-0.43761
0.351682	-0.01856
-2.20358	-0.24485
-0.55628	0.332802
-0.55863	-0.19938
-0.36242	-0.02226
-0.25515	-1.5745
-1.09295	-0.21756
-0.33456	-0.09734
-0.41677	0.418528
-0.13139	-0.14242
-0.64653	-0.28845
-0.91909	0.22381
-2.77599	0.33461
-0.95576	-0.04725
-1.55695	-0.45751
-2.5684	-0.0605
-1.82684	-1.1848
-2.91694	-0.12889
	-0.15607
	-0.16985
	-0.76549
	-0.37863
	-1.05457
	-0.53962
	0.610661
	-0.62317
	-1.30694
	0.412375

Table S9: Raw Data Related to Figure S2B:

	WT GSC	*polα50*$^{+/-}$ GSC	WT SG	*polα50*$^{+/-}$ SG
EdU+	41	50	36	47
EdU-	138	189	106	127

Table S10: Raw Data Related to Figure S2C:

Control	*polα*$^{+/-}$
4	1
2	1
1	2
6	2
2	2
1	1
2	4
7	4
1	5
1	4
3	3
3	5
5	5
2	2
1	4
3	1
1	2
0	1
2	

Table S11: Raw Data Related to Figure S2D:

Control	*polα*$^{+/-}$
1.306167	1.278792
1.228094	0.597433
1.945768	0.62422
1.875058	0.271302
0.407838	0.876815
0.620971	0.983212
0.690614	0.462132
0.561664	0.796703
0.330651	1.460722
0.492726	2.05893
0.697857	0.323502
2.412161	1.170753
0.641872	0.820372
0.354694	1.031888
0.888311	0.230859
0.65803	0.536586
1.236091	0.820468
1.260342	0.959619
0.90852	0.521959
0.48414	0.724333
1.016876	1.918949
1.09415	1.84562
0.692481	0.382616
1.179122	1.099732
0.694694	0.459023
1.33954	0.728471
1.104447	0.237306
1.101271	0.626562
0.822489	2.359881
0.604814	0.553715
1.342523	0.527155
1.473494	0.717982
0.920645	0.877052
1.552115	0.97617
0.726739	0.357884
0.954615	1.073172
1.378417	1.715323
	0.487893
	0.480964

	0.660628

Table S12: Raw Data Related to Figure S2E:

	GSC	SG
ssDNA-negative	7	10
ssDNA-positive	1	6

Table S13: Raw Data Related to Figure S3B:

Vehicle			10µM inhibitor		
All	GSC	SG	All	GSC	SG
1.668691	1.668691	2.396766	0.84715	0.84715	0.333533
1.474706	1.474706	0.997311	0.468082	0.468082	0.717872
0.663436	0.663436	0.778951	0.913295	0.913295	0.915349
0.619614	0.619614	1.131039	1.010732	1.010732	0.499032
0.548401	0.548401	0.524847	0.896314	0.896314	0.933974
0.621188	0.621188	1.074823	1.122412	1.122412	0.788468
0.802231	0.802231	1.308933	1.176643	1.176643	0.856668
0.541212	0.541212	2.200179	0.662204	0.662204	1.0727
2.010098	2.010098	1.574814	0.800588	0.800588	1.231558
0.68165	0.68165	0.574147	0.395775	0.395775	2.706675
0.673228	0.673228	0.921375	0.464453	0.464453	1.51086
0.770802	0.770802	2.422512	0.38119	0.38119	0.456647
0.478627	0.478627	0.771008	0.507934	0.507934	0.463084
0.647893	0.647893	0.500128	2.04454	2.04454	0.610301
0.497868	0.497868	0.658575	1.276271	1.276271	0.343735
0.696372	0.696372	0.864337	0.333533		0.590923
2.396766		0.593457	0.717872		0.769296
0.997311		1.310782	0.915349		2.059125
0.778951			0.499032		0.937397
1.131039			0.933974		1.706214
0.524847			0.788468		2.035227
1.074823			0.856668		1.869454
1.308933			1.0727		1.566666
2.200179			1.231558		0.841261
1.574814			2.706675		
0.574147			1.51086		
0.921375			0.456647		
2.422512			0.463084		
0.771008			0.610301		
0.500128			0.343735		
0.658575			0.590923		
0.864337			0.769296		
0.593457			2.059125		
1.310782			0.937397		
			1.706214		
			2.035227		
			1.869454		
			1.566666		

			0.841261		

Table S14: Raw Data Related to Figure S3D:

Vehicle		10μM inhibitor	
GSC	SG	GSC	SG
0.02	0.27	0.2	0.03
0.32	0.07	0.2	0.4
0.29	-0.13	0.09	0.14
0.04	-0.24	0.25	0.25
-0.03	-0.05	-0.06	0.11
0.22	0.39	0.24	-0.28
0.23	0.2	-0.13	0.1
0.2	0.01	0.07	-0.21
0.13	-0.02	0.17	0.32
0.18	-0.02	0.16	-0.28
0.06	0.01	0.18	-0.06
-0.05	0.02	0.06	0.17
0.04	-0.14	0.22	0.46
0.12	-0.08	-0.02	0.29
	-0.23	0.08	0.24
	0.05		-0.09
	-0.06		-0.07
	-0.16		
	-0.11		
	-0.14		
	0.11		

Table S15: Raw Data Related to Figure S3F:

Vehicle		10μM inhibitor	
GSC	SG	GSC	SG
0.94234	1.458179	1.043107	1.065966
0.754329	1.54104	1.04406	1.09111
1.147113	1.385793	0.555841	0.951673
1.120445	1.353411	1.010534	0.79795
0.943102	1.360268	0.887289	0.886527
1.03244	1.413795	1.116635	1.109968
1.339505	1.406557	0.83681	1.460274
0.306875	1.29055	1.206736	0.797569
1.265787	1.190354	1.103301	1.334743
1.148066	1.320266	0.696992	0.953388
	0.671467	0.712041	1.046536
	0.998152	1.46637	0.843096
	1.690954	0.756043	1.404652
	1.176448	0.671276	0.177915
	1.304836	1.309408	0.961007
	1.308456		1.228261
	1.012439		0.756424
	1.444083		0.599844
	1.323695		0.612987
	1.086348		1.011486
	1.233785		
	1.452654		
	1.561994		
	1.260262		
	1.494562		
	1.356649		
	1.079681		
	1.324457		

Table S16: Raw Data Related to Figure 3F:

nos-Gal4		*bam-Gal4*		*nos-Gal4; bam-Gal80*	
WT	*polα50*$^{+/-}$	WT	*polα50*$^{+/-}$	WT	*polα50*$^{+/-}$
0.833755	1.71324	0.023199	-0.25949	3.012224	1.130421
1.205462	1.680616	0.002493	0.614043	1.520095	1.889422
0.067381	1.085999	0.913725	1.229249	0.963087	1.55281
2.185072	1.620811	0.557483	0.831694	0.845611	0.938152
0.596007	1.004243	-0.09516	1.557561	0.674583	1.519162
1.240573	1.134904	0.64262	1.30331	1.358367	0.997146
2.138097	0.538452	0.534351	1.387124	2.503381	1.289368
1.509625	1.377369	-0.39413	0.413369	1.484098	0.700357
1.578043	0.209853	0.243138	2.71423	0.998465	0.66006
-0.70103	1.277605	0.177005	0.927196	0.769789	1.754169
0.720501	0.165427	0.535789	0.02529	2.527987	2.32908
0.441195	2.37251	1.919735	0.590838	0.647042	0.938002
0.055739	1.188624	0.067541	1.190953	2.353069	2.784909
-0.92427	1.429034	0.509258	1.138515	2.198496	1.103144
0.628984	2.772322	0.753873	1.336803	1.563693	2.021303
0.870491	3.005586	0.761944	2.862607	1.608681	2.376666
0.460992	2.465316	-0.45322	0.426856	1.735522	1.520804
1.861391	1.284234	0.810568	0.29905	1.803227	0.642919
1.048597	1.706529	-0.50049	1.812977	2.339137	1.529886
0.120114	1.52534	1.389966	3.308225		1.460099
0.916928	1.517268	1.361768	-0.05976		1.396838
2.28547	1.403463		1.058061		1.719726
-0.25156	1.667895		1.422233		1.744161
2.853039	1.914565				1.669027
0.400734					
2.197903					
1.424744					

Table S17: Raw Data Related to Figure 4E:

Control		polα50+/-	
GSC	SG	GSC	SG
1.051	0.581	1.868	1.409
1.948	0.21	0.539	1.825
1.07	0.658	1.358	1.233
0.966	0.554	0.814	1.41
1.588	0.507	0.787	1.692
0.959	0.565	0.607	0.842
1.491	0.215	1.33	2.127
1.166	0.129	1.992	2.262
1.859	0.515	0.79	1.478
0.679	0.369	2.284	1.312
1.376	0.37	0.792	0.747
2.8	0.201	0.975	
0.676		1.616	
0.831			
0.557			

Table S18: Raw Data Related to Figure S4B:

nos-Gal4 relative expression			
	Testis 1	Testis 2	Testis 3
GSC	1	0.930712	1
GB	0.771986	1	0.965367
2-cell SG	0.925594	0.632471	0.789276
4-cell SG	0.641036	0.734639	0.600625
8-cell SG	0.596229	0.620937	0.574162

bam-Gal4 relative expression			
	Testis 1	Testis 2	Testis 3
GSC	0	0	0
GB	0	0.016474	0
2-cell SG	0.186506	0.082372	0.00276
4-cell SG	0.347403	0.194399	0.438473
8-cell SG	1	1	1

nos-Gal4ΔVP16; bam-Gal80 relative expression			
	Testis 1	Testis 2	Testis 3
GSC	1	0.883686	1
GB	0.968259	1	0.709542
2-cell SG	0.968259	0.745044	0.223784
4-cell SG	0	0	0.04642
8-cell SG	0	0	0

Table S19: Raw Data Related to Figure S4E:

nos-Gal4	*bam-Gal4*	*nos-Gal4ΔVP16; bam-Gal80*	*in Silico* *nos-Gal4ΔVP16*; *bam-Gal80* and *bam-Gal4*
0.833755	0.023199	3.012224	-0.50049
1.205462	0.002493	1.520095	-0.45322
0.067381	0.913725	0.963087	-0.39413
2.185072	0.557483	0.845611	-0.09516
0.596007	-0.09516	0.674583	0.002493
1.240573	0.64262	1.358367	0.023199
2.138097	0.534351	2.503381	0.067541
1.509625	-0.39413	1.484098	0.177005
1.578043	0.243138	0.998465	0.243138
-0.70103	0.177005	0.769789	0.509258
0.720501	0.535789	2.527987	0.534351
0.441195	1.919735	0.647042	0.535789
0.055739	0.067541	2.353069	0.557483
-0.92427	0.509258	2.198496	0.64262
0.628984	0.753873	1.563693	0.647042
0.870491	0.761944	1.608681	0.674583
0.460992	-0.45322		0.753873
1.861391	0.810568		0.761944
1.048597	-0.50049		0.769789
0.120114	1.389966		0.810568
0.916928			0.845611
2.28547			0.913725
-0.25156			0.963087
2.853039			0.998465
0.400734			1.358367
2.197903			1.389966
1.424744			1.484098
			1.520095
			1.563693
			1.608681
			1.919735
			2.198496
			2.353069
			2.503381
			2.527987
			3.012224

Table S20: Raw Data Related to Figure S4F:

Vehicle	10µM inhibitor
0.508784	0.76774
-0.04526	0.326391
0.391434	0.946559
-0.12276	0.311914
-0.16576	1.312
-0.50133	0.360845
-0.13854	-0.0862
0.190232	0.195973
-0.06542	-0.05299
0.371505	0.124824
0.869037	1.206165
0.003181	0.138294
0.652619	-0.16273
0.240258	0.555803
-0.31463	-0.23241
-0.21934	1.066144
0.865918	0.867556
0.661904	0.638385
0.844635	0.651938
0.484697	0.269967
0.06871	0.406126
0.146687	0.599318
0.696014	0.694007
0.175447	0.423178
0.155875	0.203779
0.186869	1.810172
0.250217	2.353426
-0.315	0.950878
0.139259	2.567467
0.454084	2.249149
-0.18303	1.169217
0.385496	1.387287
	2.383506
	2.140027
	1.457545
	0.972286
	1.402336
	0.446447

Table S21: Raw Data Related to Figure S4I:

nos-Gal4	*nos>rpa*	*polα180*$^{+/-}$
0.833755	2.601037	1.660701
1.205462	-0.01006	2.294621
0.067381	1.819792	2.100029
2.185072	2.076201	1.851209
0.596007	1.81073	1.870423
1.240573	0.444184	1.913859
2.138097	0.941755	1.541163
1.509625	1.629409	2.06779
1.578043	1.043768	1.977457
-0.70103	1.940334	0.570688
0.720501	1.542164	2.604591
0.441195	0.883738	1.23827
0.055739	3.404706	0.96408
-0.92427	1.142323	0.582147
0.628984	1.307931	0.542417
0.870491	1.755604	1.507558
0.460992	2.376969	1.631278
1.861391	1.397628	1.874469
1.048597	0.685891	3.300395
0.120114	2.149329	3.562242
0.916928	3.55259	2.217418
2.28547	0.768625	1.607232
-0.25156	1.818162	1.243226
2.853039	1.345405	0.721922
0.400734		1.470142
2.197903		1.264972
1.424744		-0.35318
		2.33985
		1.881944

Table S22: Raw Data Related to Figure 5C and Figure S5B:

Log_2(BrdU)	Log_2(EdU)
0.949727	-0.78622
3.979822	-2.37851
2.947978	-3.12338
0.975338	-0.26303
0.494109	-0.52319
1.794681	-0.69707
0.313499	-0.90752
0.264416	0.147226
0.285157	-0.40526
2.560361	-1.06054
0.377185	-0.94404
2.900464	-1.48543
0.385654	-0.24246
1.201369	-1.25831
1.076044	-0.61857
1.662965	-1.5025
1.730393	0.21864
0.171511	-0.98296
0.587198	-0.9783
0.373352	-0.69574
0.830075	-1.11346

Table S23: Raw Data Related to Figure 5D* and S5F (log_2):

nos-Gal4	*nos-Gal4; pola50+/-*	*bam-Gal4*	*bam-Gal4; pola50+/-*	*nos-Gal4ΔVP16; bam-Gal80*	*nos-Gal4 ΔVP16; bam-Gal80; pola50$^{+/-}$*
-1.64051	1.706293	0.539826	0.456845	-0.73765	-1.36482
2.612107	-3.25913	0.140349	-1.57106	-0.95258	0.75138
0.451143	0.354129	0.035564	-1.05006	-1.89181	-0.03933
-1.74333	1.783676	-1.15265	-0.75268	-1.1849	0.70204
0.546183	-1.6443	-0.31179	0.033124	2.108614	-1.28129
-2.88245	-0.65438	0.959769	0.805337	-1.90253	-1.04057
-3.72782	-2.72158	-0.0864	-2.00483	-1.708	-0.73152
-1.44057	-3.32288	-0.47096	-2.517	-1.46108	-3.05472
-3.62121	-0.39184	0.849896	-0.83188	-4.2996	-2.8483
-2.73246	-0.87832	-0.03163	-0.31128	-1.11643	-2.33536
0.096365	-0.95884	0.505262	-1.53716	0.266644	-3.35477
0.701662	-1.14961	-0.1108	-0.42537	-0.20308	-1.95236
2.49744	0.442487	0.371136	0.274047	-1.95157	-1.36273
-0.17693	-2.98338	0.388789	-1.43808	1.353261	-1.56238
0.415777	-0.97708	0.515224	-0.99389	-0.96038	0.908932
0.63151	-1.30605	-0.28391	-0.84444	-1.6619	-1.53928
-0.7567	1.10949	0.62232	-0.5031	2.020612	-2.43426
-0.99991	1.181099	-1.15803	-1.97743	-1.48487	-3.2655
-1.29837	1.226325	-0.03333	-1.83919		0.490629
0.530978	-1.62792	-0.69519	-1.66125		-1.31617
-1.07129	-2.34696	-1.28691	-0.6374		-0.80856
-0.36712	-1.43265		-0.91854		
0.237249	-1.42746		1.550994		
-1.4912	-1.52134				
1.076845	-3.97101				
-0.37702	-2.85406				
0.469742					

*Fig. 5D displays the absolute value of the log_2 (agnostic to strandedness) and Fig. S5F displays the normal log_2 values (with respect to strandedness).

Table S24: Raw Data Related to Figure S5A:

Symmetric<br>(EdU/EdU fiber)	Weak Asymmetry<br>(EdU/BrdU less than 2-fold asymmetry)	Strong Asymmetry<br>(EdU/BrdU with greater than 2-fold asymmetry)
31	12	9

Table S25: Raw Data Related to Figure S5C:

Strong asymmetry toward the leading strand (≥ 2-fold)	Weak asymmetry toward the leading stand (< 2-fold)	Weak asymmetry toward the lagging stand (< 2-fold)	Strong asymmetry toward the lagging strand (≥ 2-fold)
3	9	4	11

Table S26: Raw Data Related to Figure S5D:

H3K27me3	PCNA	EdU
0.833755	-1.05482	-1.64051
1.205462	-1.21579	2.612107
0.067381	-0.3613	0.451143
2.185072	-0.85911	-1.74333
0.596007	-0.7166	0.546183
1.240573	-5.24247	-2.88245
2.138097	-1.49709	-3.72782
1.509625	-0.56181	-1.44057
1.578043	-2.67029	-3.62121
-0.70103	-3.0681	-2.73246
0.720501	-0.49276	0.096365
0.441195	-1.1499	0.701662
0.055739	-0.82622	2.49744
-0.92427	-0.12406	-0.17693
0.628984	-1.09902	0.415777
0.870491	-0.99608	0.63151
0.460992	-1.45258	-0.7567
1.861391	-2.06082	-0.99991
1.048597	-0.93227	-1.29837
0.120114	-1.53805	0.530978
0.916928	-0.83251	-1.07129
2.28547	-3.9494	-0.36712
-0.25156	-3.90744	0.237249
2.853039	-1.45679	-1.4912
0.400734	-0.33855	1.076845
2.197903	-1.61222	-0.37702
1.424744	-1.04414	0.469742

Table S27: Raw Data Related to Figure S5I:

	Soma	Germline
Bidirectional	10	17
Unidirectional	2	9

Table S28: Raw Data Related to Figure S5L:

Pola	Pole
0.581	0.531
4.038	0.6
7.409	0.497
1.922	0.368
2.366	1.269
2.908	0.698
3.641	0.822
0.983	0.727
1.577	0.776
3.312	0.316
3.657	0.908
1.293	0.309
2.333	0.223
8.184	0.161
	0.147
